# Modern Perspectives on Inguinal Hernia Repair: A Narrative Review on Surgical Techniques, Mesh Selection and Fixation Strategies

**DOI:** 10.3390/jcm14144875

**Published:** 2025-07-09

**Authors:** Anca Tigora, Petru Adrian Radu, Dragos Nicolae Garofil, Mircea Nicolae Bratucu, Mihai Zurzu, Vlad Paic, Raluca Gabriela Ioan, Valeriu Surlin, Dragos Margaritescu, Silviu Constantin Badoiu, Florian Popa, Victor Strambu, Sandu Ramboiu

**Affiliations:** 1 Tenth Department of Surgery, Faculty of Medicine, University of Medicine and Pharmacy “Carol Davila”, 050474 Bucharest, Romania; anca.tigora@drd.umfcd.ro (A.T.); dragos.garofil@umfcd.ro (D.N.G.); mircea.bratucu@umfcd.ro (M.N.B.); mihai.zurzu@drd.umfcd.ro (M.Z.); vlad.paic@drd.umfcd.ro (V.P.); florian.popa@spcaroldavila.ro (F.P.); victor.strambu@umfcd.ro (V.S.); 2 Department of Gynecology, Faculty of Midwifery and Nursing, University of Medicine and Pharmacy “Carol Davila”, 050474 Bucharest, Romania; raluca.ioan@umfcd.ro; 3 Sixth Department of Surgery, General Surgery Clinic I, Craiova Emergency Clinical Hospital, University of Medicine and Pharmacy of Craiova, 200642 Craiova, Romania; valeriu.surlin@umfcv.ro (V.S.); dragos.margaritescu@umfcv.ro (D.M.); sandu.ramboiu@umfcv.ro (S.R.); 4 Second Department of Morphological Science, Discipline Antomy, Faculty of Medicine, University of Medicine and Pharmacy “Carol Davila”, 050474 Bucharest, Romania; silviu.badoiu@umfcd.ro

**Keywords:** inguinal hernia repair, minimally invasive surgery, TAPP, TEP, prosthetic mesh, mesh fixation methods

## Abstract

Inguinal hernia repair is one of the most commonly performed surgical procedures worldwide, with over 20 million cases annually. The evolution of hernia surgery has transitioned from tension-based techniques to tension-free approaches, significantly reducing recurrence rates. This review explores the history, advancements, and current trends in minimally invasive inguinal hernia repair, focusing on laparoscopic techniques such as transabdominal preperitoneal (TAPP), totally extraperitoneal (TEP), single-incision laparoscopic surgery (SILS), and robotic-assisted repair. The importance of prosthetic meshes is emphasized, detailing their mechanical properties, pore size, weight classifications, and biocompatibility. Additionally, various mesh fixation methods—including tacks, sutures, and glues—are analyzed, with a discussion on their impact on postoperative complications such as chronic pain, adhesions, and infection risk. The debate between TAPP and TEP techniques is examined, highlighting the ongoing quest to determine the most effective approach. Emerging advancements, including drug-loaded meshes and dual-layered prosthetics, aim to improve integration and reduce complications. Despite significant progress, no universally superior technique or mesh exists, underscoring the need for individualized surgical approaches. Future research should focus on optimizing materials, refining fixation strategies, and enhancing patient outcomes in minimally invasive hernia repair.

## 1. Introduction

Inguinal hernia repair is among the most frequently performed elective general surgical procedures worldwide, with global estimates exceeding 20 million operations annually [1,2]. In countries with well-established healthcare systems, such as the United States, Germany, France, the United Kingdom, and the Czech Republic, hundreds of thousands of patients undergo hernia surgery each year [1,3]. The prevalence of inguinal hernias is notably high in men, with a lifetime risk of 27% to 43%, compared to 3% to 6% in women [4,5]. Men are eight times more likely than women to develop inguinal hernias and are approximately twenty times more likely to undergo surgical repair [6]. Beyond its clinical implications, this condition has a considerable socioeconomic impact, accounting for significant healthcare expenditure and loss of productivity [7]. Importantly, the failure of primary repairs increases the complexity and cost of secondary surgeries, underlining the need for durable, standardized treatment strategies [8].

Since the late 19th century, when Edoardo Bassini introduced the first formal technique for inguinal hernia repair based on suturing the posterior wall of the inguinal canal, the field has seen continuous evolution [9]. Initially, suture-based repairs were plagued by high recurrence rates and tension-related complications. The paradigm shifted dramatically with the introduction of tension-free mesh repairs by Irving Lichtenstein in 1989, a technique that rapidly became the standard of care [10]. The evolution continued with the emergence of minimally invasive approaches in the early 1990s—most notably the transabdominal preperitoneal (TAPP) and totally extraperitoneal (TEP) techniques—which demonstrated advantages such as reduced postoperative pain, shorter hospitalization, earlier return to work, and lower recurrence rates [11].

In recent years, robotic-assisted inguinal hernia repair has emerged as a promising advancement in minimally invasive surgery [12]. This technique leverages state-of-the-art robotic platforms to provide enhanced surgical precision, superior three-dimensional visualization, and improved ergonomics for the operating surgeon. These advantages are particularly beneficial in cases involving large, recurrent, or bilateral hernias, where delicate dissection and meticulous placement of prosthetic material are essential. Moreover, robotic systems may facilitate more consistent and reproducible dissection in anatomically complex regions, potentially leading to reduced intraoperative complications and shorter learning curves for certain advanced procedures [13,14].

Concurrently, the field has seen rapid innovation in the development of mesh prostheses used for reinforcement of the abdominal wall [15]. Modern meshes are now designed to be lightweight and macroporous, which promotes better tissue integration, reduces inflammatory response, and lowers the incidence of chronic pain and foreign body sensation [16,17]. Dual-layer meshes, often combining absorbable and permanent components, aim to balance strength with biocompatibility, while biologically enhanced or bioactive meshes incorporate elements such as anti-adhesive barriers, growth factors, or antibacterial agents to minimize complications and promote healing [18]. These developments are particularly relevant for patients at a higher risk of infection or those requiring intraperitoneal mesh placement [19]. At the same time, mesh fixation techniques have evolved significantly. Traditional fixation using sutures or tacks, while effective, has been associated with postoperative neuralgia, hematomas, and other complications [20]. To mitigate these risks, newer fixation strategies such as self-gripping meshes and absorbable adhesives—like fibrin sealants or cyanoacrylic glues—have been introduced [21,22,23]. These alternatives aim to reduce tissue trauma and operative time, while maintaining sufficient mesh stability during the critical early phase of tissue ingrowth. However, the choice of fixation method remains highly variable in practice, as each option presents unique advantages and potential drawbacks in terms of recurrence, chronic pain, cost, and ease of use [24,25].

Despite these advances, the field of inguinal hernia repair continues to face unresolved challenges. There is still no consensus regarding the optimal surgical approach (open vs. laparoscopic vs. robotic), the best type of prosthetic mesh, or the most effective fixation technique. The variability in clinical practice is further complicated by differences in surgeon experience, patient anatomy, and comorbidities. Notably, most existing studies have focused on short-term outcomes, and long-term data regarding recurrence, chronic pain, mesh integration, and complication rates remain limited. Additionally, while robotic surgery is gaining traction, its cost-effectiveness and clinical superiority over laparoscopic techniques are yet to be definitively proven in large-scale comparative studies [26].

Although laparoscopic approaches such as TAPP and TEP have become widely accepted in high-income settings due to favorable outcomes and reduced postoperative morbidity, their adoption remains uneven across the globe. In many low- and middle-income countries (LMICs), access to laparoscopic or robotic platforms is limited due to infrastructural constraints, financial barriers, and lack of trained personnel. As a result, open mesh repair (OMR) continues to be the predominant technique in these regions.

Similarly, the availability and type of surgical mesh vary significantly; while lightweight, large-pore, and composite meshes are commonly used in high-resource settings, polypropylene mesh—or even reused or improvised materials—may be the only option in resource-limited areas. These disparities underscore the importance of contextualizing surgical recommendations based on regional capabilities and infrastructure (Table 1 summarizes general trends in technique adoption, laparoscopic access, and mesh availability across various world regions).

The primary aim of this narrative review is to provide an updated and detailed synthesis of the current surgical techniques utilized in the treatment of inguinal hernias, with a focus on minimally invasive approaches. These include TAPP, TEP, SIL-TEP, and robotic-assisted procedures. The review discusses their anatomical considerations, operative strategies, indications, technical features, and reported outcomes, while highlighting their relative advantages, limitations, and practical applicability in clinical settings.

The second aim of this review is to analyze the evolution and current landscape of prosthetic mesh materials and fixation methods used in inguinal hernia repair. This includes a detailed evaluation of mesh types based on pore size, weight, composition, and bioactivity, as well as fixation strategies such as sutures, tacks, fibrin sealants, and self-gripping meshes. Particular attention is paid to their biomechanical performance, integration with host tissue, complication profiles, and implications for recurrence, chronic pain, and patient quality of life. By synthesizing both technical and material aspects of hernia surgery, this review aims to support evidence-based decision-making and individualized treatment planning for optimal patient outcomes.

## 2. Methodology

This review was developed to provide a comprehensive overview of current surgical approaches, mesh types, and fixation techniques used in inguinal hernia repair. A structured literature search was performed using electronic databases including PubMed, Scopus, and Web of Science. Keywords such as “inguinal hernia,” “laparoscopic repair,” “TAPP,” “TEP,” “mesh fixation,” “prosthetic mesh,” and “hernia recurrence” were used in combination with Boolean operators. The selection criteria included English-language articles published between 2000 and 2024, focusing on randomized controlled trials, meta-analyses, guidelines, and high-impact narrative reviews. Articles were screened for relevance, and data were synthesized thematically to reflect advancements in surgical technique, mesh development, and patient outcomes. Emphasis was placed on high-level evidence and studies relevant to minimally invasive surgery.

## 3. History

Hernias have been a topic of interest since the early days of surgical history [30]. The Egyptian Papyrus of Ebers, dating back to approximately 1552 BC, contains one of the earliest known references to hernias, describing them as “a swelling on the surface of the belly… what comes out… caused by coughing.” [31]. It remains a matter of debate whether hernia operations were conducted during that era. Physicians in Alexandria employed tightly fitting bandages as a treatment for inguinal hernias, while a Phoenician statuette dating back to 900 BC illustrates groin hernias being treated in this manner [32]. Aulus Cornelius Celsus (?—A.D. 50), a devoted disciple of Hippocrates, was the first to provide a detailed description of hernia surgery, marking a remarkable testament to refined surgical practice within the Alexandrian tradition [31,32,33]. He implemented the dissection and excision of the hernia sac and hemostasis performed through ligature of the vessels, taking meticulous measures to preserve the testicle [32,34].

During the Middle Ages and the pre-Renaissance era, progress in the understanding and treatment of hernias was minimal, leading to a prolonged period of decline and widespread ignorance on the subject [33,35]. The Renaissance marked a resurgence of surgical knowledge, with Ambroise Paré making a notable contribution through his detailed description of hernia repair techniques in The Apologie and Treatise. He introduced the practice of reducing the hernia sac’s contents back into the abdominal cavity, followed by suturing the peritoneum using golden thread [31,36]. In 1559, Kaspar Stromayr was the first to distinguish between indirect and direct hernias and documented his surgical intervention on a strangulated scrotal hernia in his book Practica Copiosa [31,37].

In the early nineteenth century, anatomical understanding of the inguinal canal reached a significant peak. In 1793, de Gimbernat described what became known as Gimbernat’s ligament. This was followed by Cooper, who identified the superior pubic ligament and the fascia transversalis in 1804. In subsequent years, Colles described the reflected inguinal ligament in 1811, Hasselbach defined the anatomical triangle bearing his name in 1814, and Cloquet detailed the eponymous gland and the processus vaginalis in 1817 [31,38,39,40]. Despite these significant advancements in anatomical knowledge and the introduction of anesthesia in 1846, progress in hernia surgery remained limited, as sepsis continued to pose a major obstacle to safe and effective surgical intervention [41].

Edoardo Bassini of Pavia (1844–1924) revolutionized the management of inguinal hernias with his innovative surgical technique. His method involved high ligation and excision of the hernia sac, followed by reconstruction of the posterior wall of the inguinal canal using the conjoined tendon and the transversalis fascia, reinforced with a triple-layer suture [9,31]. Numerous modifications of Bassini’s original repair have been introduced over time, with surgeons around the world adapting the technique to suit their clinical practices. Notable contributors to these refinements include Henry O. Marcy, William S. Halstead, Chester McVay, and Edward E. Shouldice, each of whom played a significant role in the evolution of modern hernia repair methods [11,12,15,33,42].

The 20th century marked the beginning of the tension-free era in hernia repair, driven by growing research aimed at reducing recurrence rates. To address issues related to tension on the suture line, some surgeons introduced relaxing incisions in the anterior sheath of the rectus abdominis muscle—a technique known as the Wolfler method. This was often followed by suturing the lateral flap to Poupart’s ligament, a modification referred to as the Berger technique [31]. An alternative strategy for addressing the issue of tension in hernia repair involved the use of non-autologous materials. In 1887, Henry O. Marcy was among the first to experiment with animal-derived tissues for hernioplasty, utilizing tendons from kangaroo, ox, whale, and deer as prosthetic reinforcement materials [43]. Subsequently, homologous tissues—including the external oblique aponeurosis, fascia lata grafts from the thigh, and split-thickness skin grafts—were employed as reinforcement materials in hernia repair. However, these techniques were soon abandoned due to suboptimal outcomes and a high incidence of complications [44,45].

The introduction of mesh in hernia repair dates back to the 1950s and is credited to Dr. Francis Usher, who developed one of the first synthetic woven prostheses. He observed that tissue ingrowth through the mesh’s interstices contributed to effective reinforcement and long-term stability of the abdominal wall [46]. In the same decade, Cumberland and Scales proposed eight essential criteria for the ideal implantable biomaterial—standards that continue to influence the development and evaluation of surgical prostheses to this day (Table 2) [47,48].

In 1959, Nyhus and colleagues pioneered the use of synthetic mesh to reinforce the posterior wall of the inguinal canal via a preperitoneal approach. Later, in 1989, Irving Lichtenstein revolutionized hernia surgery by introducing the concept of tension-free prosthetic repair using mesh, which he performed under local anesthesia in an outpatient setting. This technique rapidly gained widespread adoption and became the standard of care for groin hernia repair [44]. His technique of prosthetic hernioplasty involved extraperitoneal reconstruction of the inguinal canal floor using a synthetic mesh. In a landmark study comprising 1000 patients, Lichtenstein reported no hernia recurrences over a five-year follow-up period, further establishing the efficacy and durability of the tension-free repair method [49]. In the late 20th century, the rapid advancement of laparoscopic surgery catalyzed the integration of minimally invasive techniques into a wide range of surgical procedures, including inguinal hernia repair. In 1990, Ger et al. published a pivotal study describing a transabdominal laparoscopic approach, which utilized staple fixation for mesh placement under direct visualization, marking a foundational step in the evolution of laparoscopic hernioplasty [50]. Even today, there is no universal consensus regarding the optimal technique for hernioplasty. Nevertheless, an ideal operative approach should demonstrate a low incidence of complications, be relatively easy to learn, facilitate rapid postoperative recovery, ensure consistent and reproducible outcomes, and maintain overall cost-effectiveness [5]. The current inguinal hernia repair techniques performed worldwide according to the HerniaSurge Group are summarized in Table 3.

While the chronological evolution of inguinal hernia repair demonstrates a steady accumulation of anatomical knowledge and technical innovation, its true value lies in how each milestone has influenced and shaped modern surgical principles and patient care strategies. The historical trajectory is not simply a timeline of discoveries but a reflection of surgery’s enduring attempt to balance effectiveness, safety, and simplicity.

The work of Bassini, for example, was far more than an isolated surgical technique—it introduced the concept of anatomically informed reconstruction, a principle that remains central to modern hernioplasty. His understanding of posterior wall weakness and structured tissue reinforcement foreshadowed the biomechanical focus of contemporary repair strategies. Bassini’s repair, although performed without prosthetics, laid the foundation for the layered closure concepts later incorporated into mesh-based techniques. His legacy persists today, not only in modified open repairs but also in the logic behind mesh placement planes in laparoscopic procedures.

Lichtenstein’s innovation in the late 20th century marked a philosophical shift in surgical strategy: from tension-based anatomical reconstruction to functional reinforcement using prosthetic materials. This “tension-free” concept radically reduced recurrence rates and postoperative discomfort. More importantly, it democratized hernia repair by making outpatient surgery under local anesthesia both safe and effective. These principles directly inform today’s emphasis on cost-effective, day-case procedures, particularly in healthcare systems facing resource limitations.

The rise of laparoscopic techniques in the 1990s introduced a new surgical paradigm that emphasized minimal invasiveness, faster recovery, and bilateral repair through the same access points. It also initiated a deeper dialogue around surgical learning curves, procedural standardization, and complication management. Importantly, laparoscopy highlighted the need for structured training pathways, simulation models, and credentialing systems that are now standard in surgical education. This milestone not only influenced technique but also the infrastructure of surgical education and quality control.

The recent integration of robotic systems marks yet another transformative stage. Beyond offering better visualization and precision, robotic surgery is reshaping the ergonomics of the operating room, enabling older surgeons or those with physical limitations to extend their careers. It is also pushing the field closer to digitally integrated surgery, where artificial intelligence, intraoperative data, and automated assistance may eventually refine decision-making and execution. The impact of robotics is not merely technical but conceptual: it embodies the modern goals of precision medicine, patient-centered customization, and standardization without sacrificing flexibility.

## 4. Laparoscopic Techniques

Selecting the optimal technique for inguinal hernia repair remains a subject of ongoing clinical and academic discussion. An ideal surgical approach must fulfill several key criteria: low recurrence and complication rates, a short learning curve, rapid postoperative recovery, reproducible outcomes, and overall cost-efficiency. Mesh-based repairs have become the standard of care in many settings, particularly due to their proven efficacy in minimizing recurrence rates. The 2009 European Hernia Society guidelines recommend mesh-based repair for all symptomatic inguinal hernias in adult males over 30 years of age [5].

Laparoscopic approaches emerged in the early 1990s as minimally invasive alternatives to traditional open repairs. Ger et al. were among the first to describe the laparoscopic technique, marking a pivotal advancement in hernia surgery [50]. Currently, the two most widely used minimally invasive methods are the transabdominal preperitoneal (TAPP) and totally extraperitoneal (TEP) repairs [51]. Both involve placement of a prosthetic mesh in the preperitoneal space to reinforce the abdominal wall, yet they differ significantly in anatomical access and operative technique [52].

In 1992, Arregui and colleagues introduced the TAPP method, which accesses the preperitoneal space via the peritoneal cavity. In contrast, the TEP technique, first described by Ferzli in 1993, is performed entirely within the preperitoneal space without breaching the peritoneum [53,54].

TAPP involves accessing the preperitoneal space through the peritoneal cavity, which provides surgeons with a wide operative field and enhanced the visualization of key anatomical structures such as the inferior epigastric vessels, cord structures, and hernia orifices. This makes TAPP particularly advantageous for complex or recurrent hernias and facilitates bilateral repairs within the same surgical session. Furthermore, due to the familiar intra-abdominal orientation, TAPP tends to have a shorter learning curve for surgeons with existing laparoscopic experience. However, because it involves entry into the peritoneal cavity, TAPP carries a small but notable risk of intra-abdominal complications, including visceral injury, adhesions, and postoperative ileus [55,56,57].

In contrast, TEP is performed entirely within the extraperitneal space without breaching the peritoneal cavity. This eliminates the risk of intra-abdominal organ injury and reduces the likelihood of postoperative adhesions. Additionally, TEP has been associated with lower rates of scrotal edema and seroma formation in certain populations. However, it presents technical challenges due to the confined working space and lack of natural anatomical landmarks. TEP also demands greater precision in dissection and port placement, making it more technically demanding, particularly during the early phase of a surgeon’s learning curve [58,59]. Prior lower abdominal surgery, obesity, and large scrotal hernias can further complicate this approach. Early complications such as subcutaneous emphysema and conversion to TAPP or open surgery are more commonly reported during the initial learning phase [51].

A systematic review by Basukala et al. (2024) [60], encompassing 23 randomized controlled trials with over 2200 participants, concluded that there is likely little to no difference between TAPP and TEP in terms of serious adverse events (0.4% vs. 0.7%) and recurrence rates (1.2% vs. 1.1%). The review noted very low certainty of evidence regarding chronic pain outcomes and perioperative complications such as hematoma or seroma. Notably, TEP was associated with a higher conversion rate to TAPP or open surgery (2.5% vs. 0.7%), suggesting a steeper learning curve for the extraperitoneal approach [60]. Similarly, Ozel et al. (2024) [61] found that while TAPP resulted in significantly fewer early postoperative complications and shorter hospital stays, it carried a risk of serious intraoperative injuries, including bowel perforation and major bleeding. In contrast, the TEP group experienced a higher rate of urinary retention and surgical emphysema, highlighting its technical difficulty [61]. Dokania et al. (2023) also reported that TEP offered superior pain control in the early postoperative period and facilitated faster recovery, but at the cost of longer operative times and higher conversion rates due to peritoneal breach [62].

From a cost-effectiveness perspective, laparoscopic hernia repairs—whether TAPP or TEP—typically incur higher initial expenditures due to the need for general anesthesia, specialized equipment, and longer operative times, particularly in the early stages of surgeon training [60]. Nevertheless, these costs are frequently offset by downstream savings, including shorter hospitalizations, fewer wound-related complications, and faster return to daily activities [63]. The evidence suggests that, in bilateral or recurrent hernias especially, laparo-endoscopic approaches are economically advantageous, as they allow for simultaneous bilateral repairs through a single operative session with minimal additional morbidity. Furthermore, both TAPP and TEP have been associated with lower rates of chronic postoperative groin pain compared to open techniques—an outcome that has important long-term economic and quality-of-life implications [64].

Both TAPP and TEP are safe and effective approaches with similar long-term outcomes. TAPP may be favored in anatomically complex cases or among less experienced surgeons due to easier visualization, while TEP offers the advantage of avoiding peritoneal entry but demands greater technical skill. Current evidence does not clearly favor one approach over the other, and the choice of technique should be tailored based on patient-specific anatomy, surgeon expertise, and institutional resources. Importantly, when considering both clinical outcomes and economic impact, laparoscopic approaches provide significant advantages over open surgery in appropriately selected patients [51,54,65].

## 5. TAPP Surgical Technique

**Figure 1:**
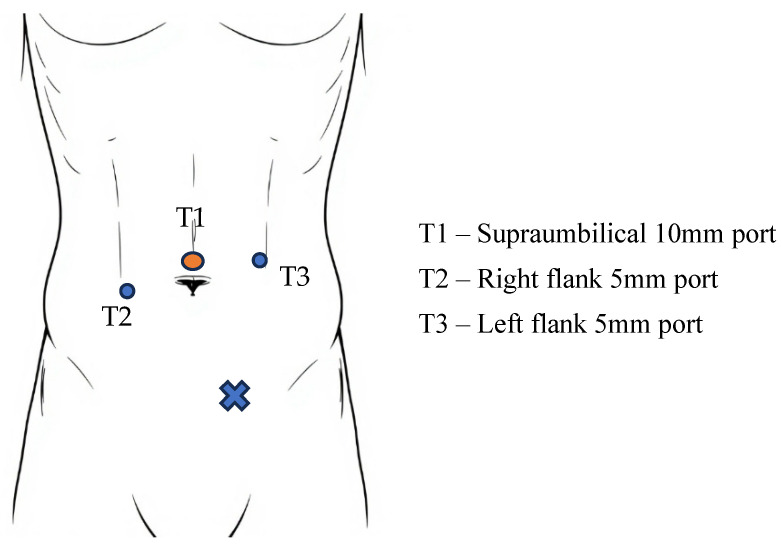
Port placement for unilateral left inguinal hernia repair. Modified from reference [43].

The procedure begins with an incision made 2–5 cm above the umbilical scar. Depending on the specific clinical scenario, access to the peritoneal cavity is achieved using either a Veress needle or an open (Hasson) technique to establish a pneumoperitoneum. Subsequently, a 10–12 mm trocar (T1) is inserted, followed by the introduction of a 30-degree laparoscope [66]. After inspecting the lower abdomen, the subsequent 5 mm trocars (T2 and T3) are strategically placed on the transverse umbilical line, 6–8 cm lateral to T1, all while carefully avoiding the inferior epigastric vessels [67]. For unilateral hernia repairs, the ipsilateral port is placed 1 cm above the level of the umbilicus, while the contralateral port is positioned 1 cm below [66] (Figure 1).

Once properly positioned, the small intestine should be gently maneuvered away from the surgical field. If the hernia defects contain any viscera, these should be carefully reduced by either direct grasping or applying manual pressure to the external abdominal wall. In cases where adhesions are present, proper viscerolysis is necessary in order to alleviate any weight on the peritoneal flap, facilitating a clearer surgical view and improving the overall procedure [66].

Creating a large peritoneal flap is essential for properly exposing the entire myopectineal orifice and ensuring accurate mesh placement. Initially, an incision is made in the peritoneum approximately 2 cm above the anterior superior iliac spine. With the non-dominant hand, the peritoneum is gently retracted downward and medially, allowing CO_2_ to fill the preperitoneal space, which facilitates pneumodissection and separation of the tissue planes. The incision continues horizontally from lateral to medial until it reaches the medial umbilical ligament [68].

**Figure 2:**
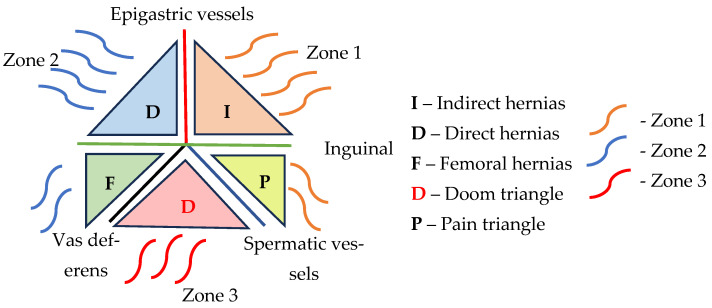
Illustration of the “five triangles” in right inguinal hernia. Modified from reference [34].

The dissection area is systematically divided into three zones based on the posterior anatomy of the inguinal region. This anatomical classification is conceptually framed by the “Inverted Y,” the “Three Zones,” and the “Five Triangles,” as described by Furtado et al. [34] (Figure 2). This structured approach aims to ensure that, in every case, the nine procedural steps detailed by Daes and Felix are thoroughly completed [69].

Zone 1 is situated lateral to the inferior epigastric and spermatic vessels. The anterior limit of this dissection is defined by the anterior superior iliac spine (ASIS), while the posterior boundary is the psoas muscle. It is crucial to maintain the dissection anterior to the preperitoneal fat to prevent injury to the lateral femoral cutaneous nerve, genitofemoral nerve, and branches of the iliohypogastric nerve, all of which traverse this area. Inadvertent nerve injury can result in neuropathic pain or paresthesia. Furthermore, careless dissection in this zone may damage the gonadal vessels, increasing the risk of hematoma or testicular atrophy. Proper placement of the inferolateral corner of the mesh is essential here to prevent indirect hernia recurrence and mesh folding, which could lead to chronic discomfort or recurrence of symptoms [66].

Zone 2 is located medial to the inferior epigastric vessels and is associated with the region of direct hernias. Dissection in this zone should adequately expose the Retzius space, ensuring identification of the pectineal (Cooper’s) ligament and pubic symphysis. Although this region contains loose areolar tissue, blunt dissection must be performed carefully to avoid bladder injury, especially in patients with large direct hernias or previous pelvic surgery. Over-dissection or improper mesh fixation in this area may result in osteitis pubis, pubic pain syndrome, or hematoma, particularly if the mesh tacks are placed too close to the pubic bone. Extending the dissection 1–2 cm beyond the midline is necessary to provide sufficient space for mesh overlap, reducing the likelihood of medial recurrence [34].

Zone 3 represents the most technically demanding and high-risk area, often referred to as the “triangle of doom” due to the presence of critical vascular structures, including the external iliac artery and vein. This zone requires mobilization of the peritoneum over the vas deferens and spermatic vessels to fully dissect the indirect hernia sac. Complications in this zone include vascular injury, which may result in life-threatening bleeding, and inadvertent damage to the vas deferens, potentially compromising fertility in younger patients. Excessive traction on the hernia sac may also lead to nerve entrapment or chronic orchialgia. Given the high density of essential structures, a meticulous technique and clear anatomical visualization are imperative in this zone to prevent intraoperative complications and recurrence [34,66,68].

Once the preperitoneal space has been thoroughly dissected, positioning a large prosthetic mesh becomes straightforward. The mesh should adequately cover all areas of potential weakness in the inguinal region. Medially, it should extend to at least the pubic symphysis; laterally, it should reach the iliopsoas muscle. Inferiorly, it must descend 1–2 cm below the pubis, while superiorly, it should cover the anterior abdominal wall and extend 3–4 cm beyond the hernial defect [34,70]. The subsequent step involves securing the mesh, for which several fixation methods are documented in the literature. These include staple fixation, the application of fibrin glue, the use of self-gripping mesh, transabdominal sutures, and the more recent no-fixation technique [70]. Similar techniques are employed to close the peritoneal flap, adhering to the following guidelines: avoid bone structures, protect the inferior epigastric vessels, evade the triangles of disaster and pain, and limit the number of fixation points [34].

The TAPP approach can theoretically be applied to nearly all types of hernias, including those that are strangulated or incarcerated, with few exceptions. However, the decision to use this technique ultimately depends on the surgeon’s clinical judgment and expertise. The indications and contraindications for this procedure are outlined in the following section (Table 4).

## 6. TEP Surgical Technique

**Figure 3:**
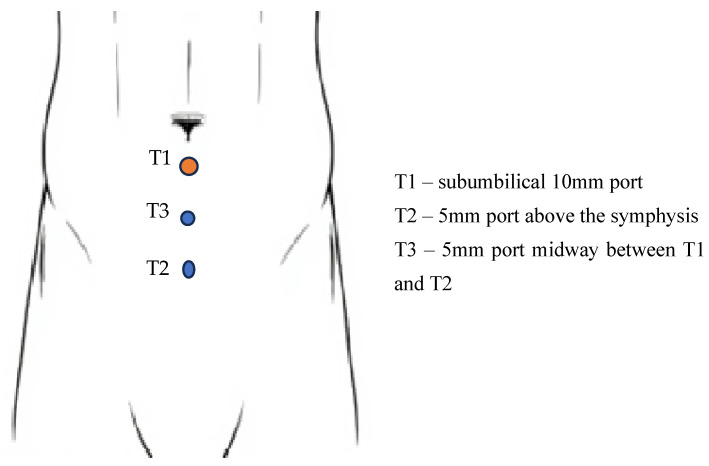
Port placement TEP procedure.

The procedure begins with an incision made directly below the umbilicus, followed by dissection of the subcutaneous tissue until the anterior sheath of the rectus muscle is reached. Once the sheath is opened on the side corresponding to the hernia, the rectus muscle is gently retracted laterally to expose the posterior sheath of the rectus muscle [71]. The next step involves the placement of a dilation balloon into the preperitoneal space, directed toward the pubic symphysis. The balloon is then gradually inflated while carefully observing the expansion of the inguinal space [71]. A 10–12 mm trocar (T1) is inserted, and the preperitoneal space is insufflated to 10 mmHg using carbon dioxide. Subsequently, a 10 mm, 30-degree laparoscope is introduced to further expand the preperitoneal space, facilitating the accurate placement of the additional 5 mm trocars [68]. The two additional trocars are positioned along the midline under direct visualization. A 5 mm trocar (T2) is placed just above the pubic symphysis, while the other (T3) is inserted midway between this trocar and the camera port [71] (Figure 3).

The mesh should extend medially to the pubic symphysis and laterally to the anterior superior iliac spine and the iliopsoas muscle. Inferiorly, it needs to extend 1 to 2 cm below the pubic bone, while superiorly, it should cover 3 to 4 cm of the anterior abdominal wall in relation to the deep inguinal ring. There are various methods available to secure the mesh; however, in the majority of hernia cases, fixation is typically unnecessary [71].

While various procedural variations exist in laparoscopic inguinal hernia repair, Dr. George S. Ferzli proposed a standardized and systematic approach to the TEP technique, known as the “Seven Rules” or “Seven Steps.” Introduced to enhance procedural safety and reproducibility, these steps provide a logical framework that allows for anatomical consistency, minimization of complications, and optimal mesh placement. Their origin lies in Ferzli’s extensive clinical experience, which demonstrated that adherence to a precise sequence of dissection improves outcomes and simplifies the learning curve for surgeons transitioning to minimally invasive techniques [72].

**The Seven Steps by Ferzli**

1. **Identify the pubic symphysis**
  The pubic symphysis serves as a central anatomical landmark and reference point for subsequent dissection. Correct identification ensures a midline orientation and facilitates symmetrical mesh positioning.
2. **Blunt dissection of Cooper’s ligament bilaterally**
  This exposes the retropubic (Retzius) space and defines the medial limit of dissection. Careful dissection here helps avoid vascular injury and ensures that the mesh has firm anchorage on both sides, minimizing medial recurrence.
3. **Identify Hesselbach’s triangle and the three hernia orifices (direct, femoral, obturator)**
  This critical step ensures that all potential sites of herniation are visualized and adequately covered. It promotes a comprehensive repair and prevents future defects by enabling a thorough anatomical assessment.
4. **Identify and elevate the inferior epigastric vessels**
  Elevation of these vessels exposes the underlying space of Bogros laterally and prevents inadvertent vascular injury. Awareness of this anatomy is key to preventing bleeding and facilitates proper lateral dissection.
5. **Develop the space of Bogros laterally up to the anterior superior iliac spine (ASIS)**
  This step allows full lateral exposure for appropriate mesh coverage, especially critical in indirect hernias. It helps prevent folding or underlap of the mesh, which could lead to recurrence.
6. **Dissect the spermatic cord (or round ligament in females)**
  Proper identification and gentle dissection of these structures help avoid trauma to the vas deferens, testicular vessels, or surrounding nerves, reducing the risk of infertility, hematoma, or chronic pain.
7. **Place a large mesh with adequate overlap**
  The mesh should cover all anatomical defects with sufficient overlap to prevent recurrence. It should extend medially over the pubic symphysis, laterally to the ASIS, and inferiorly over the femoral canal, with minimal fixation to reduce nerve irritation and postoperative pain.

Ferzli’s structured method addresses many of the anatomical and technical challenges unique to the extraperitoneal approach. By ensuring each zone is dissected in a defined sequence, it minimizes the chance of incomplete hernia repair, improper mesh deployment, and iatrogenic complications such as bleeding, nerve injury, or visceral trauma. The approach is particularly beneficial during the learning curve, where standardization improves the consistency and outcomes across varying levels of surgical experience [51,71].

## 7. Complications

Laparoscopic or robotic inguinal hernia repair shares several complications with open techniques, such as hematoma, seroma, and recurrence, but also introduces specific risks unique to the minimally invasive approach. Reported complication rates vary widely in the literature, ranging from less than 3% to as high as 20%, depending on patient factors, surgeon experience, and procedural complexity [73]. As surgeons gain experience with the technique, they can identify and correct early complications, leading to a steady decline in their incidence over time [74]. Another study conducted by Felix et al. analyzed 1423 laparoscopic inguinal hernia repairs performed over a six-year period. The authors observed a significant reduction in complication rates—from 5.6% in the first three years to just 0.5% in the latter half of the study. Notably, 90% of the complications occurred within the first half of the cases, underscoring the critical influence of the surgical learning curve on outcomes and patient safety [75]. Laparoscopic herniorrhaphy complications can be broadly categorized into intraoperative and postoperative events. A detailed overview of adverse outcomes specifically associated with the TAPP and TEP techniques is presented in Table 5 [73,74].

Intraoperative complications typically stem from technical errors, such as the improper use of mechanical or thermal energy, or misidentification of anatomical structures and their spatial relationships within the operative field. Adhering to fundamental surgical principles—such as adequate exposure, careful tissue handling, and meticulous hemostasis—remains essential for minimizing risk and ensuring procedural success.

Bowel injury represents one of the most serious intraoperative risks, particularly during procedures involving peritoneal entry. This risk is heightened in cases where the small bowel is involved in the hernia sac or adherent due to chronicity, prior pelvic surgery, or previous mesh placement. According to published data, the incidence of intestinal injury during TAPP repair is approximately 0.09%. In contrast, this complication is even less common in the TEP approach, as the procedure avoids breaching the peritoneal cavity [76]. Thermal injury poses a significant challenge during laparoscopic hernia repair, as it is often not immediately apparent intraoperatively and may result in delayed bowel perforation. Consequently, the use of energy-based devices near the intestine should be minimized or avoided when possible to mitigate this risk [76]. If a bowel injury is identified during the procedure, laparoscopic repair may be considered, provided the surgeon has adequate experience. In the absence of such expertise or in the case of extensive injury, conversion to an open surgical approach may be required. Moreover, when significant contamination is present, mesh implantation should be avoided to minimize the risk of infection and postoperative complications [77].

Another potential iatrogenic intraperitoneal injury is bladder damage, which necessitates prompt laparoscopic primary repair using absorbable sutures, followed by continuous bladder drainage with an indwelling catheter to ensure proper healing and prevent urinary leakage [78]. Extraperitoneal bladder rupture has been reported during the TEP procedure, particularly during balloon dissection following initial entry into the preperitoneal space. Additionally, the placement of secondary trocars inferior to the camera port poses a risk of bladder injury if not performed with caution and under direct laparoscopic visualization [77]. A large series of laparoscopic inguinal hernia repairs reported an incidence of bladder injury occurring in approximately 1 out of every 650 procedures [79]. Mesh placement may still be considered in cases without evidence of bacterial colonization or clinical signs of urinary tract infection. To ensure the integrity of the bladder repair, a cystogram is typically performed 1–2 weeks postoperatively to assess for any leakage before removing the indwelling catheter and surgical drain [77].

During inguinal hernia repair, it is essential to identify and preserve several key vascular structures, including the iliofemoral, inferior epigastric, gonadal, and “corona mortis” vessels, to minimize the risk of intraoperative bleeding. Particular caution is warranted when dissecting near the iliofemoral vessels—especially in the presence of large direct or femoral hernia sacs, or when prior mesh placement has altered the local anatomy [73]. Injuries to the epigastric vessels during inguinal hernia repair are typically managed intraoperatively with hemostatic clips. However, if bleeding is delayed and results in significant hematoma within the preperitoneal space, selective arterial ligation becomes more complex. In such cases, open surgical intervention may be necessary, often employing the Stoppa technique to achieve adequate hemostasis and exposure [44]. 

Vascular injury may also occur during mesh fixation, particularly when tacks are placed too close to major vessels. This risk can be mitigated by ensuring that all fixation devices are applied above the iliopubic tract, thereby avoiding the underlying vascular structures in the so-called “triangle of doom” [80]. The incidence of postoperative hematomas following TAPP surgery has been reported at approximately 3.4%. While vascular injuries appear to be more commonly associated with TEP procedures than with TAPP, the overall reoperation rates between the two techniques remain comparable according to current literature [76].

Lateral dissection during TAPP surgery exposes several critical nerves within the so-called “triangle of pain,” including the ilioinguinal, iliohypogastric, genitofemoral, femoral, and lateral femoral cutaneous nerves. Injury to any of these nerves may result in chronic postoperative groin pain, significantly affecting the patient’s quality of life [73], which occurs in approximately 0.5% to 4.6% of cases, depending on the repair technique used and the surgeon’s level of expertise [81]. While local anesthetic infiltration can provide temporary relief from nerve-related pain, effective management hinges primarily on prevention. This includes meticulous dissection and careful identification of anatomical landmarks. Additionally, peritoneal tears may occur during lateral dissection, and improper placement of mesh fasteners—especially near the obturator foramen—can result in nerve injuries, further emphasizing the need for a precise technique and anatomical awareness [76]. As surgical knowledge of nerve anatomy from the abdominal perspective has improved, the incidence of postoperative neuralgia has progressively declined, reflecting the benefits of enhanced anatomical awareness and refined dissection techniques [74].

The contents of the spermatic cord—including the vas deferens, testicular arteries and veins, lymphatic vessels, and associated nerves—are enclosed within a multilayered myofascial extension of the abdominal wall. Due to their close proximity to both indirect and direct hernia defects, these structures are particularly vulnerable to injury during inguinal hernia repair [77]. Rough or careless handling of these delicate structures can result in bleeding from the testicular or cremasteric vessels, potentially leading to hematoma formation, postoperative orchitis, or, in severe cases, testicular atrophy [73]. Transection of the vas deferens may occur during dissection and, if identified in younger patients, should be managed with an end-to-end anastomosis to preserve fertility. In older individuals, simple ligation or clipping is generally considered safe. Although such injuries occur in less than 1% of hernia repairs, they can result in significant long-term complications, including reduced fertility and sexual dysfunction [77,82].

Hypercapnia, along with related complications such as subcutaneous emphysema and pneumomediastinum, is a recognized risk during laparoscopic procedures, often occurring beyond the surgeon’s direct control. These complications are more frequently associated with the extraperitoneal approach compared to other techniques. To reduce the incidence of such events, maintaining lower insufflation pressures may be advantageous [73].

Postoperative urinary retention is a relatively common complication following inguinal hernia repair, with reported incidence rates ranging from 1.3% to 5.8%. This condition may result in delayed hospital discharge, increased patient discomfort, a heightened risk of urinary tract infections, and potential urethral trauma [74,81]. Several factors have been associated with the development of postoperative urinary retention, including male gender, advanced age, the type of anesthesia administered, and postoperative pain management strategies. Male patients, in particular, exhibit a higher incidence of acute urinary retention following various surgical procedures; however, this association appears less consistent in the context of minimally invasive inguinal hernia repairs. Furthermore, both general and regional anesthesia have been implicated in increasing the risk of urinary retention, likely due to their inhibitory effects on the autonomic nervous system and suppression of voluntary micturition reflexes [77]. Decisions regarding perioperative catheterization should be individualized, taking into account the patient’s specific risk factors, surgical context, and overall clinical status [74].

Recurrence of inguinal hernias following surgical repair remains a significant concern for both patients and surgeons. While it is not always classified as a direct complication, it nonetheless represents a failure of the intervention and underscores the importance of a meticulous technique, appropriate mesh selection, and adequate anatomical coverage [77]. Preventing hernia recurrence is paramount, as reoperations are associated with increased technical complexity, higher complication rates, and a greater physical and psychological burden for the patient. When evaluating the risk of recurrence, both surgeon-dependent and patient-related factors must be taken into account. Patient-specific preoperative predictors include an advanced age, female gender, body mass index (BMI) greater than 30 kg/m^2^, smoking, diabetes mellitus, connective tissue disorders, a chronic cough, long-term steroid use, and a history of oncological treatments. These comorbidities may impair wound healing, alter intra-abdominal pressure dynamics, or compromise tissue integrity, all of which contribute to a higher likelihood of recurrence [83]. Intraoperative causes of hernia recurrence are multifactorial and often stem from technical shortcomings during the procedure. Inadequate dissection is a common issue, potentially resulting in missed hernia defects, unrecognized lipomas of the cord, or incomplete reduction in the hernia sac—each of which can lead to recurrence. Similarly, improper mesh placement remains a significant risk factor. This includes the use of a mesh that is undersized, incorrectly shaped, or poorly oriented within the preperitoneal space, thus failing to adequately cover all potential defect sites. Insecure fixation or insufficient overlap can result in mesh migration, rolling, or folding, especially under intra-abdominal pressure. Additionally, complications such as postoperative hematoma or seroma formation can disrupt mesh integration and positioning, further contributing to long-term failure of the repair. Therefore, a meticulous surgical technique and attention to anatomical detail are essential to minimize these risks. These issues highlight the critical importance of a precise technique, meticulous dissection, and careful handling of the mesh to ensure the optimal placement and fixation, thereby minimizing the risk of hernia relapse [84].

## 8. Prosthetic Meshes

The utilization of meshes in inguinal hernia repair has significantly lowered the rates of recurrence, which has shifted the focus away from hernia relapse as the primary outcome measure after surgery [85]. Currently, increased attention is being directed toward other adverse outcomes, such as acute and chronic postoperative pain, as well as the overall duration of recovery following the procedure [86]. The properties of the mesh play a critical role, as they significantly influence the fibrotic tissue response, the incidence of chronic pain, the flexibility of the abdominal wall, and other postoperative outcomes [87]. The mechanical properties of the prosthesis—including the material, pore size, weight, biocompatibility, and propensity for shrinkage—play a crucial role in determining how effectively the mesh integrates with the surrounding tissue during the healing process. [88].

For optimal healing, it is essential that the hernia mesh mimics the tension and elasticity of the abdominal wall, allowing it to withstand the physiological pressures generated during daily activities. Abdominal wall tension can be estimated using Laplace’s law, which describes the relationship between tension, pressure, wall thickness, and diameter in an elastic spherical structure [89].

$$\mathrm{Tension}\;=\frac{Diameter\times Pressure}{4\times Wall\;thickness}$$

This formula provides a mathematical framework for understanding how these factors interact, enabling a better grasp of the mechanical demands placed on the abdominal wall. In a healthy adult, the peak intra-abdominal pressure is usually generated during coughing or jumping and can reach around 170 mmHg [90]. When applying Laplace’s law in this context, the theoretical maximum tensile strength required of a hernia mesh is estimated to be around 32 N/cm for large hernias and 16 N/cm for smaller defects [91]. Given that the abdominal wall demonstrates approximately 38% elasticity at a tension of 32 N/cm, it is therefore recommended to utilize implant materials that possess lower resistance and enhanced elasticity to better mimic the biomechanical properties of native tissue [87,89].

Porosity refers to the proportion of open space relative to solid material in a mesh, which can be expressed by volume, area, or weight. Among the parameters influencing mesh behavior, pore size is particularly critical, as it directly impacts the host tissue response and determines the degree of cellular infiltration, vascularization, and integration of the implant into surrounding tissues [91]. In 2008, Earle and Mark proposed a classification system for hernia mesh based on pore size, as outlined in Table 6. Meshes with pore diameters greater than 0.075 cm facilitate rapid infiltration by fibroblasts and macrophages, thereby promoting early collagen deposition and the formation of connective tissue that secures the mesh within surrounding structures. Moreover, larger pore sizes allow better penetration by immunocompetent cells, improving the local immune response and reducing the risk of mesh-related infections [89]. On the other hand, meshes with excessively large pore sizes may increase the risk of adhesions to adjacent viscera, potentially leading to serious complications such as mesh erosion and the development of enterocutaneous or enterovesical fistulas [87]. When meshes with pore sizes smaller than 0.8 mm are implanted, a rigid layer develops at the implantation site, leading to a reduction in flexibility and potentially hindering the normal function of surrounding tissues. This occurs because individual granulomas develop around each mesh fiber and subsequently fuse together, creating a cohesive layer that envelops the mesh [92]. Meshes with larger pores (greater than 1 mm) promote greater soft tissue in-growth and exhibit enhanced flexibility due to reduced granuloma bridging [91].

The weight of the mesh is determined by both the polymer weight and its pore size. Considering the current products and available research, an optimal overall density for the mesh is likely between 28 g/m^2^ and 90 g/m^2^. Earle and Mark classified mesh weights into four distinct categories, as presented in Table 7 [93].

Heavyweight meshes, which possess thick polymers, small pore sizes, and high tensile strength, weigh over 90 g/m^2^, with mesh their substantial material mass contributing to their strength [87,89]. Heavyweight meshes are associated with increased instances of chronic pain, a significant foreign body reaction, enhanced fibrosis, and a higher likelihood of adhesions, which can ultimately result in greater mesh shrinkage [91]. Lightweight meshes, which feature larger pores (greater than 1 mm), are made of thinner filaments and possess greater elasticity [87]. These meshes elicit a milder foreign body reaction and, although they exhibit reduced tensile strength (minimum tensile strength is around 10 N/cm^2^) and weigh less than 5 kg/m^2^, they are capable of withstanding pressures exceeding the maximum abdominal pressure [89].

The selection of mesh weight in inguinal hernia repair has been extensively studied for its potential impact on postoperative outcomes, particularly chronic pain and patient quality of life. A randomized clinical trial by O’Dwyer et al. compared lightweight and heavyweight meshes in inguinal hernia repairs. The study found that 39.5% of patients with lightweight mesh reported pain of any severity at 12 months postoperatively, compared to 51.6% in the heavyweight mesh group. However, the recurrence rate was higher in the lightweight group (5.6% vs. 0.4%), suggesting a trade-off between pain reduction and recurrence risk [95]. 

Conversely, a study by Melkemichel et al. found no significant difference in chronic pain rates between lightweight and heavyweight meshes, with 15.8% and 16.2% of patients reporting pain, respectively. Notably, a younger age was identified as a significant risk factor for chronic pain, irrespective of mesh type [96]. 

Further supporting this, a multicenter randomized controlled trial by Bona et al. found that at 6 months post-surgery, 25% of patients reported pain of some intensity, with severe pain reported by 1% of patients in both lightweight and heavyweight mesh groups. Notably, the use of lightweight mesh was associated with a statistically significant reduction in pain at 1 week and 6 months postoperatively (*p* = 0.02 and *p* = 0.04, respectively), as well as improved quality of life scores at 1 and 6 months (*p* = 0.05 and *p* = 0.02, respectively) [97]. A meta-analysis by Bakker et al. concluded that heavyweight meshes should be used in laparo-endoscopic repairs of direct or large inguinal hernias to reduce recurrence rates, as lightweight meshes provided no significant benefit in preventing chronic pain [98].

The composition of a mesh significantly influences the rate of infection following implantation [87]. Meshes are primarily categorized into three types based on their composition: monofilament, multifilament fibers, and patches (CPTFE) [89]. Multifilament meshes pose a greater risk of infection as bacteria can become ensnared in the smaller pores, hindering the ability of immune cells to access and eliminate these pathogens [92].

**Figure 4:**
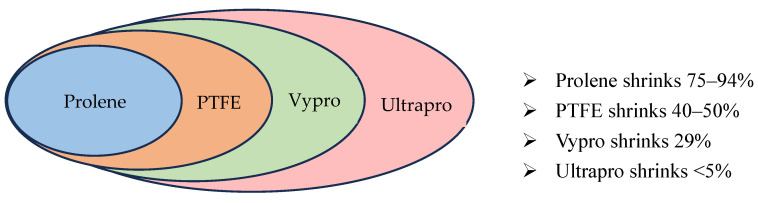
Shrinkage properties of meshes [63].

Shrinkage refers to the contraction of the mesh that occurs as scar tissue accumulates during the tissue remodeling process. This contraction exerts tension on the margins of the hernia defect, which may lead to loosening of the mesh and, ultimately, recurrence of the hernia [87]. Heavyweight meshes with small pore sizes are particularly susceptible to shrinkage due to the formation of a dense scar plate, which can significantly compromise the long-term stability and integration of the repair [19,86,87,89]. The shrinkage properties of different mesh types are summarized Figure 4.

A diverse range of synthetic meshes is available on the market today. Presently, three biomaterials are predominantly used globally for laparoscopic hernia repair: polyester mesh, polypropylene mesh, and the expanding (e)-PTFE patch [99]. The first generation of synthetic meshes can be broadly categorized into three distinct types: macroporous meshes, microporous meshes, and macroporous meshes incorporating a multifilament or microporous component [100]. While non-absorbable synthetic materials provide the necessary mechanical strength to support hernia repair, they are also associated with increased inflammation, immune response, and reduced elasticity. These biological reactions can contribute to mesh shrinkage, which in turn heightens the risk of adhesion formation [91]. Despite the clinical approval of first-generation synthetic meshes, their use has been associated with several complications, including hernia recurrence, infections, adhesions, and chronic pain. For instance, a review published in Polymers highlights that complications such as mesh stiffness over time, hernia recurrence, mesh erosion, and adhesions have been documented with non-absorbable synthetic meshes [46]. Additionally, a study in Frontiers in Surgery notes that issues such as inflammation, wound healing problems, postoperative and chronic pain, seromas, adhesions, migration of the mesh, and implant rejection have been encountered following mesh implantation [18]. To address these complications, a second generation of meshes was created by integrating various synthetic and biological components, enhancing the properties of the mesh for improved performance [87].

Prosthesis are categorized based on material, composition, pore size, and other factors, all of which are effectively addressed in Table 8 [89,101].

## 9. Fixation Methods

Applying a mesh to strengthen the posterior wall of the inguinal canal requires the use of a fixation material to anchor it onto the defect and prevent its migration during the early postoperative period [87]. In both open and laparo-endoscopic hernia repair, securing synthetic mesh involves weighing the strength of fixation against the risk of local tissue trauma and nerve damage from entrapment [5]. There are several methods available for mesh fixation, including glues (fibrin sealant or cyanoacrylic glue), tacks, sutures, or self-adhesive options. Nevertheless, there is no consensus on the optimal binding method; thus, the methods employed often reflect the preferences of individual surgeons and personal experience of the occurrence of complications. Common complications associated with mesh repair include chronic pain, fluid accumulation, adhesions, erosion into solid or hollow organs, and mesh failure, which may involve shrinkage, detachment, or migration, leading to repair malfunction [102].

Fibrin sealant (FS) gained clinical approval in the United States in 1998 and has been commercially available in Europe for over two decades [103,104]. FS is a biodegradable adhesive formed through the combination of human-derived fibrinogen and thrombin activated by calcium chloride. This results in the formation of polymerized fibrin chains, effectively mirroring the final step of the coagulation cascade [105]. It has been employed as a potent hemostatic agent in cardiothoracic and trauma surgery, to bolster high-risk gastrointestinal anastomoses, prevent pancreatic fistulas, and address air leaks following thoracic procedures [106]. Apart from its hemostatic function, the fibrinogen component contributes to the product’s tensile strength and adhesive properties, while the thrombin component facilitates fibroblast proliferation [105]. These characteristics prompted its application in hernia repair, with its initial introduction in 2001 by Katkhouda et al. in an experimental study conducted on pigs [106].

Cyanoacrylic (CA) glues are hybrid tissue sealants made of synthetic materials that find widespread use in the food and drug industry, household activities, and medicine [22,107]. N-butyl-2-cyanoacrylate has emerged as the preferred tissue adhesive due to its benefits, including efficient and instantaneous hemostasis, bacteriostatic properties, slow degradation, reduced toxic effects, and rapid and strong bonding to both soft and hard tissues [108]. CAs operate through a mechanism similar to the granulation process observed at different stages of wound healing, and its applications in surgery span a broad spectrum, including organ, vessel, skin, or mucosa repairs [109,110] (Table 9).

Tack fixation has been utilized since the introduction of laparoscopic repairs for inguinal and ventral hernias in the late 1980s to early 1990s [111]. In contemporary surgical practice, various types of mesh fixation tacks are commonly employed. These may be helical or specially contoured in design and are generally categorized as either permanent (typically composed of titanium or non-titanium alloys) or absorbable, depending on their composition and intended duration of fixation [112]. Tacks are simple to apply, being inserted perpendicularly into the abdominal wall with a penetration depth ranging from 3.7 to 6.7 mm [113]. Tacks provide strong fixation but their use is linked to significant morbidity, including nerve and vessel entrapment, inflammation, pain, adhesion formation, or tack migration [111,114].

ProTack, the first tack ever produced, remains the most widely used device in clinical practice [111]. This laparoscopic instrument is specifically designed to insert a titanium coil into the fascia and muscle layers of the anterior abdominal wall [115]. The tack itself boasts a distinctive helical shape, measuring 4 mm in length and 3 mm in width, and it penetrates the underlying tissues to a depth of approximately 3 to 4 mm. For optimal effectiveness and secure placement, it is crucial that the tacks are positioned 1 to 1.5 cm apart along the periphery of the mesh to ensure proper fixation while avoiding complications [116].

PermaFix is designed as a permanent helical hollow fastener featuring an atraumatic tip to minimize tissue damage during insertion and is constructed from a molded polymer material known as polyacetal. The overall length of the tack is 6 mm, with the head contributing an additional 0.7 mm [115].

Currently, absorbable tacks (AbsorbaTack, PermaSorb, and SorbaFix) are associated with reduced rates of inflammation, adhesion formation, and migration, meaning the use of titanium tacks is less recommended. These tacks are composed of polymers or copolymers, such as poly(D,L)-lactide or glycolide-co-L-lactide, measuring between 6.4 and 6.7 mm, and they are designed to be absorbed within 12 to 16 months [87,111] (Table 10).

Sutures employed in laparoscopic hernia repair are classified into two categories: absorbable and non-absorbable. Each type is distinguished by the level of tension it exerts and the duration of strength retention before degradation occurs [111] (Table 11). Absorbable sutures are also categorized as natural or synthetic. Natural sutures are derived from animal tissues, which degrade through enzymatic proteolysis, leading to a poor tensile strength and unstable performance. In contrast, synthetic sutures, made from materials like polyglycolic acid or polydioxanone, provide greater tensile strength, reliability, and controlled absorption, breaking down through hydrolysis for more consistent degradation [117]. Sutures are classified as monofilament or multifilament, each with unique characteristics. Monofilament sutures, made of a single strand, pass through tissues smoothly with minimal inflammation but have higher memory, making knot security more challenging [118]. Multifilament sutures, composed of braided or twisted fibers, are more pliable and easier to handle, with superior knot security, but their increased surface area raises the risk of friction, inflammation, and infection [119].

## 10. Discussion

The direction in general surgery is the minimally invasive approach to pathologies, aiming to minimize trauma and ensure rapid postoperative recovery, enhancing patient comfort and facilitating quick reintegration into society. The SIL-TEP repair is a minimally invasive surgical technique for inguinal hernia repair that combines the single-incision approach with the totally extraperitoneal method, avoiding entry into the peritoneal cavity [120]. Since Cugura JF et al. first documented a case of single-incision SIL-TEP in 2008, multiple studies have since validated its safety, feasibility, and effectiveness [121]. Research has demonstrated that SIL-TEP offers comparable outcomes to conventional multi-port laparoscopic techniques, with potential benefits such as reduced postoperative pain, improved cosmetic results, and faster recovery times [122]. Robotic inguinal hernia repair, first introduced in the urologic literature and later in general surgery by Dominguez et al. in 2015, has emerged as an alternative to traditional minimally invasive techniques by enhancing precision, dexterity, and visualization [123]. Although robotic-assisted surgery is often praised for its high-resolution 3D visualization, tremor elimination, and instrument stability, which are thought to enhance surgeon comfort and performance, studies do not support these advantages in the context of minimally invasive inguinal hernia repair [124]. A randomized clinical trial involving 102 patients, comparing robotic and laparoscopic minimally invasive inguinal hernia repair, found no significant differences in early clinical outcomes, including postoperative pain, quality of life, ergonomics, cosmetic results, wound morbidity, and complications. Moreover, the robotic approach proved to be more expensive, required longer operative times, and led to greater surgeon frustration [12]. A study conducted by Huerta et al., which analyzed data from 1299 patients, compared operative times among open, laparoscopic, and robotic techniques for hernia repair. The findings indicated that, relative to the open approach, operative times were 19% longer for laparoscopic techniques and 79.3% longer for robotic procedures. Additionally, when comparing the two minimally invasive techniques, the robotic approach had a 49% longer operative time than the laparoscopic method [11]. In a separate study, Waite compared 24 laparoscopic hernia repairs with 39 robotic hernia repairs, finding no recurrence or inguinodynia, with longer operative times for the robotic technique but faster recovery compared to the laparoscopic approach [125]. Additionally, a retrospective multicenter study by Kolachalam comparing 93 open hernia repairs with 95 matched-control robotic-assisted repairs in obese patients found similar 30-day outcomes between the two groups, with fewer postoperative complications in the robotic group [126]. Charles et al. conducted a single-institution retrospective review comparing 191 open, 241 laparoscopic, and 69 robotic primary unilateral inguinal hernia repairs, finding similar rates of adverse events, readmissions, and mortality across all groups [127]. By integrating AI, robotic systems can enhance precision in surgery by analyzing clinical data to predict complications and recurrence risks, thus enabling more tailored and accurate surgical planning in line with the growing trend of precision medicine and personalized treatment approaches. Moreover, AI can optimize the use of robotic systems in tailoring personalized approaches for hernia repair, considering individual patient factors like anatomy and genetic background, potentially leading to improved outcomes [128].

While robotic-assisted inguinal hernia repair offers increased dexterity, 3D visualization, and improved ergonomics, its cost-effectiveness remains limited—especially in comparison to conventional laparo-endoscopic methods. A multicenter cost analysis in the U.S. demonstrated that r-TAPP incurs significantly higher fixed costs—including device acquisition and maintenance—resulting in an average total cost of USD 5517 vs. USD 3269 for TAPP alone [129,130]. Similarly, a meta-analysis found the operative time and hospital costs to be higher across most studies. Though some case series indicate shorter hospital stays and lower complication-related charges with the robotic approach, these benefits do not fully offset the increased OR and equipment expenses [131].

In LMICs, where healthcare budgets are constrained and laparoscopic infrastructure is already limited, the high capital and ongoing costs of robotic platforms are even less justifiable. Therefore, TEP and TAPP remain more feasible and cost-effective alternatives, offering similar clinical outcomes with significantly lower financial burden. Economic evaluations should include not just direct costs but also indirect factors—such as return-to-work time, complication avoidance, and the learning curve—while recognizing that r-TAPP may be appropriate in high-resource or complex-case settings [130].

The debate between TEP and TAPP repair dates back to the earliest descriptions of these techniques and has since become a key topic in abdominal wall surgery. Over the past two decades, numerous systematic reviews have been published, reflecting the growing interest in optimizing minimally invasive hernia repair [51]. In 2020, a systematic review by Hung et al. analyzed data from 14 trials, comparing 659 TEP patients and 682 TAPP patients based on several postoperative outcomes, including seroma formation, edema, hematoma, intraoperative injury, urinary retention, epigastric vessel bleeding, and wound infection. The study found that TEP had a higher seroma rate than TAPP, while TEP showed lower scrotal/cord edema rates both immediately postoperatively and one week after surgery. Other outcomes showed no significant differences between the techniques [132]. In 2021, Aiolfi et al. conducted a systematic review comparing TEP and TAPP, analyzing data from 15 randomized controlled trials involving 1359 patients. The study found no significant differences between TEP and TAPP in hernia recurrence, chronic pain, operative time, wound complications, hospital stay, resuming professional activities, and costs [133]. Concerning intraoperative complications, visceral injuries after TAPP have been reported in 0.3–0.6% of cases versus 0.2–0.1% in TEP, while vascular injuries are slightly more frequent with TEP (0.4%) than TAPP (0.1–0.2%) [134].

Despite significant advancements in hernia repair, the question of “Which mesh is superior?” remains unanswered, as each type comes with its own advantages and limitations, preventing the establishment of a clear consensus on the optimal choice for surgical use [87]. Non-absorbable synthetic meshes offer excellent mechanical strength, but they are also associated with chronic inflammation, postoperative pain and adhesions due to mesh shrinkage [91]. Polypropylene meshes (PP) are cost-effective and integrate well with the host tissue, but they are also the most commonly linked to chronic pain [135]. Polyester meshes (PET) are less commonly used due to conflicting opinions regarding their clinical performance. Studies suggest that PET meshes exhibit greater cytocompatibility, as they provoke less inflammation and foreign body response, resulting in improved tissue integration and favorable histological properties. However, a notable drawback is their increased susceptibility to infections over time, raising concerns about their long-term safety and effectiveness [136]. Compared to PP and PET meshes, expanded polytetrafluoroethylene (ePTFE) meshes offer lower adhesion rates but suffer from poor tissue integration, which is linked to a higher risk of hernia recurrence [87]. Clinical studies reported a 4.4% recurrence rate and 44% incidence of comorbidities among patients with ePTFE implants [137], in contrast to low adhesion formation documented in 91% of cases [138].

To reduce complications associated with traditional meshes, a new generation of dual-surface meshes was developed, featuring different properties on each side by combining various materials. The visceral-facing side is designed to be smooth and microporous, minimizing the risk of adhesions, while the opposite side, in contact with abdominal tissue, is rough and macroporous to promote tissue integration [139]. These meshes are typically built on a polypropylene (PP) backbone, enhanced with synthetic or natural polymers to improve biocompatibility and anti-adhesive properties, offering a more balanced solution for hernia repair [46]. Multiple non-adhesive coatings such as chitosan, cellulose, collagen, omega-3 fatty acids, or mussel-derived copolymers are used in composite mesh fabrication to minimize adhesions and improve biocompatibility, though their effectiveness varies [87].

A recent advancement in the field is the development of drug-loaded meshes, designed to address the ongoing clinical challenges of infection and inflammation by incorporating therapeutic agents directly into the mesh structure [140]. In 2019, Perez-Kohler et al. conducted an experimental study on the use of a chlorhexidine-loaded carboxymethylcellulose gel as a coating for hernia repair prosthetic materials, which concluded that antibacterial gel-coated PP meshes effectively prevent bacterial adhesion without negatively impacting wound healing [141]. PP meshes have also been infused with rifampicin, hexamethylene diisocyanate, or β-cyclodextrins to enhance their antimicrobial properties [140]. These loading techniques expand possibilities beyond antibiotics, allowing the incorporation of growth factors and cytokines to enhance healing [87]. In 2019, Wang et al. conducted a study which developed a bFGF-loaded mesh that inhibited inflammation, promoted fibroblast proliferation, improved collagen expression, and regulated immune-related cytokines, facilitating tissue regeneration [142]. Nanotechnology has advanced drug delivery by enhancing the solubility, bioactivity, and targeting of therapeutic agents. In this context, natural plant compounds, known for their diverse pharmacological properties, can be integrated into nanostructures to overcome challenges like poor bioavailability [143]. Micro/nanorobots are widely used in biomedical applications such as drug delivery, surgery, and biosensing, enabling targeted drug release. Biomimetic robots, derived from natural cells like erythrocytes and neutrophils, can inherit biological features that help them navigate complex in vivo environments. Red blood cell-based microrobots, for instance, offer long circulation times and large cavities, making them ideal for intelligent drug delivery systems [144]. As a vision for the future of medical treatments, integrating natural plant compounds into drug-loaded meshes through nanotechnology or using natural cell nanorobots can optimize controlled release and targeted delivery, further enhancing the meshes’ effectiveness and improving therapeutic outcomes.

There is no consensus on the optimal fixation method, and the choice largely depends on surgeon preference. However, strong evidence supporting a specific method that enhances patient outcomes or surgical success could significantly influence clinical practice [5]. A systematic review of 12 RCTs involving 1992 primary inguinal hernia repairs evaluated mesh fixation methods and reported chronic pain rates ranging from 0% to 36.3% and no significant impact on infection rates. The overall chronic pain rates associated with different mesh fixation methods were 14.7% for sutures, 7.6% for n butyl-2 cyanoacrylate glue, 3.7% for fibrin sealant, and 18.2% for self-fixing meshes [145]. In a meta-analysis, Sajid et al. concluded that staple fixation is associated with a significantly higher incidence of chronic pain compared to glue fixation [146]. In terms of relapse, a study from the Danish Hernia Database analyzed 1535 patients and found no significant difference in long-term reoperation rates or clinical recurrence between fibrin sealant and tacks for mesh fixation in TAPP inguinal hernia repair over a median follow-up of 31 months [147].

Fibrin sealants have gained favor in laparoscopic groin hernia repair due to their biocompatibility and ability to mimic natural tissue healing processes. A study by Helvind et al. demonstrated that mesh fixation using fibrin sealant significantly reduces the long-term reoperation rate compared to mechanical fixation with tacks (0.89% vs. 2.94%, *p* = 0.031), indicating that fibrin sealants not only promote early tissue integration but also support durable mesh stabilization with minimal long-term complications. The favorable outcomes associated with fibrin sealants can be attributed to their gradual bioresorption and replacement with organized collagen fibers, enhancing the physiologic integration of the mesh into the abdominal wall [148]. In contrast, cyanoacrylate adhesives provide robust and immediate mechanical fixation through rapid polymerization upon tissue contact. Research published by Pascual et al. in 2018 [149] found that cyanoacrylate adhesives, despite eliciting a more pronounced macrophage response compared to traditional sutures, ultimately achieved optimal tissue integration and sustained mechanical strength over an extended period. The chronic inflammatory response triggered by cyanoacrylate adhesives, characterized by macrophage infiltration and subsequent fibrous encapsulation, may thus represent a double-edged phenomenon: on one hand, ensuring stable and strong mesh fixation over time, and on the other, raising concerns about potential excessive fibrosis, compromised elasticity, and chronic pain [149].

Collectively, these findings highlight the complexity inherent in choosing adhesive fixation methods for hernia repair. While fibrin sealants offer biological advantages through natural tissue remodeling, cyanoacrylate adhesives provide a superior initial fixation strength at the potential cost of prolonged inflammatory responses. Clinicians must therefore balance immediate mechanical needs with long-term biological implications, taking into account patient-specific factors such as predisposition to chronic inflammation, tissue elasticity, and potential for fibrosis.

The choice of suture material for mesh fixation in inguinal hernia repair remains an area of ongoing research, especially concerning its impact on postoperative complications such as chronic pain and recurrence. While non-absorbable sutures have historically been preferred due to their tensile strength and durability, recent evidence suggests that absorbable sutures may offer comparable outcomes in both open and laparoscopic approaches, with potential benefits in terms of postoperative comfort and a reduced foreign body response. Agarwal et al. conducted a prospective observational study evaluating the impact of non-absorbable (prolene) versus absorbable (vicryl) sutures on postoperative chronic groin pain following Lichtenstein hernioplasty. Their findings demonstrated no statistically significant difference in the incidence or severity of chronic groin pain at six months, suggesting that the suture type may not be the primary determinant of postoperative discomfort, and reinforcing the importance of other intraoperative factors such as nerve handling and mesh position [150].

In the context of laparoscopic repair, Bilaloglu’s 2024 [151] study compared early complications between absorbable and non-absorbable fixation materials in patients undergoing TAPP repair. The study found no significant differences in rates of pain, scrotal edema, seroma, or urinary retention between the two groups during early follow-up intervals (1 week, 1 month, and 6 months), reinforcing the safety of absorbable devices in minimally invasive hernia surgery [151]. Furthermore, a meta-analysis by Khan et al. compared absorbable versus non-absorbable tacks in laparoscopic ventral hernia repair and similarly reported no statistically significant differences in recurrence rates, postoperative pain, or infection. These findings support the equivalency of both fixation approaches in laparoscopic hernia surgery, though they emphasize the need for more long-term studies beyond 24 months [152]. Additionally, a systematic review by Sajid et al. evaluating ten randomized controlled trials on absorbable versus non-absorbable sutures found no significant difference in operative morbidity or infection rates, while absorbable sutures were associated with a significantly lower risk of wound dehiscence (OR = 0.12; 95% CI: 0.04–0.39; *p* < 0.0004) [153]. This suggests a potential advantage of absorbable sutures in certain clinical contexts.

Taken together, these studies suggest that absorbable sutures and fixation devices offer comparable clinical outcomes to their non-absorbable counterparts in both open and laparoscopic hernia repairs. While long-term randomized controlled trials remain warranted to solidify these findings, current evidence supports their safe and effective use, especially in patients where reducing chronic pain and foreign-body reaction is a priority.

The implantation of a surgical mesh is a well-established procedure, but surgery itself is only the initial phase of the healing process, because the alloplastic material triggers a complex biological response involving both hematological and tissue mechanisms [87]. The body’s immediate reaction includes protein absorption, complement system activation, coagulation, platelet aggregation, and neutrophil activation—all of which play a crucial role in managing inflammation and initiating repair. Simultaneously, tissue-level responses such as cell proliferation, adhesion, and fibrosis contribute to the long-term integration of the mesh, ultimately influencing the success of the procedure and the patient’s recovery [154]. Research indicates that in patients with recurrent hernias, collagen metabolism is disrupted in three key ways: a reduced collagen I-to-III ratio (with a higher proportion of weaker type III collagen), lower collagen quality (thinner fibers contributing to structural weakness), and increased collagen degradation (elevated matrix metalloproteinase-2 activity, which preferentially breaks down type I collagen) [155,156]. Mesh complications arise from multiple factors, so solely focusing on mesh development may not be sufficient to resolve postoperative issues. Patient-related factors, including an advanced age, female gender, BMI over 30 kg/m^2^, smoking, diabetes, connective tissue disorders, chronic cough, steroid use, and prior chemotherapy, have been shown to impact postoperative complications in hernia repair [157].

As multiple surgical options exist for inguinal hernia repair, each with distinct benefits and limitations, a structured comparison is valuable for clinical decision-making. The selection of technique should be tailored to the patient’s clinical profile, the surgeon’s experience, and the available resources. Table 12 offers a concise comparison of the most commonly used techniques—including open anterior mesh repair (Lichtenstein)**,** laparoscopic TAPP, TEP, SIL-TEP, and robotic-assisted approaches—and their advantages and disadvantages.

Long-term follow-up (>5 years) is essential for assessing the durability and quality of inguinal hernia repairs. Meta-analyses show that recurrence rates for laparoscopic (TEP/TAPP) and open mesh techniques are similarly low (~1.8–2.1%) at 5–10 years, with open approaches having higher rates of chronic pain (up to ~15%) compared to laparoscopic methods [162]. Lightweight mesh in both open and laparoscopic repairs is associated with a significantly reduced incidence of chronic pain and foreign-body sensation without compromising long-term hernia recurrence rates [163]. A large prospective cohort study evaluating the outcomes of TEP repair using partially absorbable mesh demonstrated excellent long-term efficacy. Specifically, the study reported a remarkably low recurrence rate of just 0.67% over a five-year follow-up period. Notably, no cases of chronic postoperative pain were observed among the patients, suggesting that partially absorbable mesh may offer significant benefits in minimizing long-term complications while maintaining surgical durability [164]. Likewise, robotic-assisted repairs (r-TAPP) demonstrate durable results with minimal recurrence (~0.5%) and low chronic pain, although long-term data remain more limited [165]. Table 13 summarizes these long-term outcomes by surgical technique and mesh type.

Beyond the surgical technique, optimizing postoperative analgesia plays a central role in minimizing complications and enhancing recovery after open inguinal hernia repair. Postoperative pain management following open inguinal hernia repair is a critical component of enhanced recovery. Comparative evidence supports the use of multimodal analgesia, typically combining NSAIDs (such as ibuprofen or diclofenac) and acetaminophen, which together provide superior pain relief and reduce the need for opioids compared to monotherapy [166]. When opioids are required, short-term and rescue dosing is recommended due to risks of nausea, sedation, and dependency. Local anesthetic infiltration, especially with agents like bupivacaine, significantly reduces early postoperative pain and delays the need for systemic analgesia. Additionally, ilioinguinal and iliohypogastric nerve blocks may further improve outcomes, although they require appropriate expertise and their benefit over local infiltration remains debated [167,168]. Among available regimens, NSAID–acetaminophen combinations with local anesthetic infiltration are considered most effective and well-tolerated for standard open repairs [168].

To enhance clinical applicability, we propose a structured approach to guide the selection of the most appropriate inguinal hernia repair technique. This decision-making process should consider three core dimensions: patient factors, hernia characteristics, and surgical context.

First, patient-related factors such as age, comorbidities, and ASA score help determine the suitability for general anesthesia and tolerance of more invasive procedures. In elderly or high-risk patients who may not tolerate general anesthesia well, open mesh repair remains a preferred option, particularly in settings lacking laparoscopic infrastructure.

Second, the type of hernia plays a critical role. For patients presenting with bilateral or recurrent hernias, laparoscopic techniques such as TEP or TAPP are generally recommended, as they allow simultaneous bilateral repair and offer lower postoperative pain and faster recovery.

Third, the surgical setting and resources significantly influence the choice. In high-resource centers with robotic platforms and experienced teams, SIL-TEP or robotic TAPP may be selected, particularly for patients desiring better cosmetic outcomes or in anatomically complex cases. Conversely, in low-resource environments, where laparoscopic tools or advanced meshes are unavailable, surgeons may opt for open mesh repair or even non-mesh repair techniques in the context of contamination or infection risk.

## 11. Conclusions

Laparoscopic inguinal hernia repair techniques, particularly TAPP and TEP, have become widely accepted due to their demonstrated efficacy, reduced postoperative pain, and faster recovery compared to traditional open approaches. These minimally invasive methods offer comparable outcomes in terms of recurrence and complication rates, making them suitable alternatives in most clinical scenarios. However, the decision between TAPP and TEP should be individualized, taking into account the surgeon’s expertise, the patient’s anatomical considerations, and the technical capabilities of the healthcare institution.

An essential determinant of long-term outcomes in hernia surgery is mesh selection. Lightweight, large-pore prosthetic materials are generally associated with lower rates of chronic pain and reduced foreign body sensation, though recurrence risks must also be considered when choosing the most appropriate mesh. Equally important is adherence to precise anatomical dissection techniques, such as those guided by structured protocols like Ferzli’s seven rules, which have been shown to reduce intraoperative complications and enhance procedural safety.

The mesh fixation strategy should be carefully tailored to each patient’s individual needs. The use of atraumatic methods and absorbable tacks has been shown to decrease the risk of postoperative neuralgia and chronic discomfort. Additionally, while laparoscopic approaches may initially involve higher procedural costs, their long-term cost-effectiveness is evident—particularly in the management of bilateral and recurrent hernias—due to reduced hospital stays, fewer postoperative complications, and quicker return to normal activity.

Future directions in inguinal hernia repair should emphasize the development of improved mesh materials, the refinement of minimally invasive techniques, and the personalization of surgical strategies. Such advancements have the potential to further reduce postoperative morbidity, enhance patient quality of life, and optimize surgical outcomes across diverse patient populations.

## Figures and Tables

**Table 1:** Regional variation in access to inguinal hernia repair techniques and mesh availability based on references [5,27,28,29].

Region	Most Common Technique	Laparoscopic Access	Robotic Access	Mesh Availability
North America	TAPP, TEP, OMR	Widespread	Moderate–High	Lightweight, composite, 3D meshes
Western Europe	TAPP, TEP, SIL-TEP, OMR	Widespread	Moderate	Broad access to all mesh types
Eastern Europe	TAPP, TEP, OMR	Moderate	Low	Mainly polypropylene
Latin America	OMR, some TEP	Limited–Moderate	Rare	Limited lightweight mesh options
Sub-Saharan Africa	OMR	Rare	Very Rare	Often lacks mesh or uses reused polypropylene
South and Southeast Asia	OMR, some TAPP/TEP	Limited	Rare	Highly variable by country and institution

**Table 2:** Criteria for the ideal implantable biomaterial established in the 1950s [31].

Remain unaltered by tissue fluids	Withstand mechanical strains
Be chemically inert	Be moldable into the required form
Not induce an inflammatory or foreign body reaction	Not cause allergic reactions or hypersensitivity
Be noncarcinogenic	Be capable of sterilization

**Table 3:** Current inguinal hernia repair techniques [5].

Non-mesh techniques	▪ Shouldice ▪ Bassini ▪ Desarda
Open mesh techniques	➢ Lichtenstein ➢ Transinguinal preperitoneal (TIPP) ➢ Transrectal preperitoneal (TREPP) ➢ Plug-and-patch PHS (bilayer)
Laparo-endoscopic mesh techniques	❖ Totally extraperitoneal (TEP) ❖ Transabdominal preperitoneal repair (TAPP) ❖ Single-incision laparoscopic repair (SILS) ❖ Robotic repair

**Table 4:** Indications and contraindications of TAPP procedure. Modified from reference [48].

Indications	Contraindications
Type 3 and Type 4 hernias (Nyhus classification) Bilateral hernias Hernias in individuals with obesity Hernias occurring in those engaged in high levels of physical activity Recurrence of hernias following open surgical repairs	➢ Intolerance to capnoperitoneum ➢ Severe clotting disorders ➢ Pregnancy beyond the second trimester ➢ Recurrence of hernias following laparoscopic repairs ➢ Pediatric patients ➢ Severe ascites ➢ Strangulated or perforated hernias accompanied by concurrent sepsis Large scrotal hernias ➢ Extensive intra-abdominal adhesions ➢ Patients who have undergone radical prostatectomy

**Table 5:** Complications from laparoscopic herniorrhaphy.

Intraoperative	Postoperative
Visceral trocar injury Vascular injury Nerve Injury Cord structures injury Abdominal wall hematoma Hypercapnia Subcutaneous emphysema pneumomediastinum Pneumothorax	Seroma Hematoma Wound Infection Hydrocele Neuralgia Groin pain Testicular complications Bowel obstruction Mesh complications Urinary retention Recurrence

**Table 6:** Classification of mesh pore size [93].

Pore Type	Pore Size (mm)
Microporous	<0.10
Small pore	0.10–0.60
Medium pore	0.60–1
Large pore	1–2
Very large pore	>2

**Table 7:** Classification of mesh weight [93].

Mesh Type	Density (g/m^2^)	Advantages	Disadvantages	Ref.
Ultra-lightweight	<35	- Reduced foreign body sensation - Lower risk of chronic pain - Enhanced flexibility and patient comfort	- Potential for higher recurrence rates in large or direct hernias due to decreased mechanical strength - May exhibit increased elasticity, leading to bulging in certain cases	[87]
Lightweight	35–50	- Balanced flexibility and tensile strength - Decreased chronic pain and foreign body sensation compared to heavier meshes	- Slightly increased risk of recurrence in larger hernias - May not provide sufficient support in high-tension repairs	[94]
Medium weight	50–90	- Suitable for a broad range of hernias - Provides adequate support with moderate flexibility	- Higher foreign body reaction compared to lightweight meshes - Increased risk of chronic pain relative to lighter meshes	[92]
Heavyweight	>90	- High mechanical strength - Lower recurrence rates in complex or large hernia repairs	- Increased risk of chronic pain and stiffness - Greater foreign body reaction, leading to fibrosis and adhesions - Higher likelihood of mesh shrinkage and reduced abdominal wall compliance	[92]

**Table 8:** Type of meshes for laparoscopic groin hernia repair [18,101].

Type of Mesh	Material	Constitution	Pore Size (mm)	Properties
Totally macroporous	Polypropylene	Monofilament	>3 mm	Non-absorbable Hydrophobic Thermostable Persistent tensile strength High infection resistance
Totally microporous	Polytetrafluoroethylene (CTPTFE)	Patches	<0.06	▪ 30–40 days to integrate ▪ Reduced risk of adhesions ▪ Low infection resistance
Macroporous with microfilament or microporous components	Polyester	Plated multifilament threads	>0.075	✓ High risk of adhesions ✓ Good infection resistance
Mixed	Polypropylene + polyglecaprone-25 (Ultrapro)	Monofilament	>3 mm	➢ Partially absorbable ➢ Increased tensile strength ➢ Low tissue reaction
	Polypropylene + titanium (Ti-Mesh)	Monofilament	>1 mm	- Non-absorbable - Low tissue reaction
	Polyester + collagen layer (composite)	Multifilament	>3 mm	- Reduced risk of adhesions - Reduced shrinkage - Hydrophilic
	Polyester + polylactic acid micro-grips (Progrip)	Monofilament	>1 mm	- Immediate adherence to tissue - Difficult to maneuver

**Table 9:** Mesh fixation glues available on the market [87].

Fixation Type	Manufacturer	Material
Fibrin Glue	Tissel (Baxter, Bucharest, Romania)	Human fibrinogen, synthetic aprotinin, human albumin, L-histidine, niacinamide, polysorbate 80, sodium citrate dihydrate, human thrombin, calcium chloride dihydrate, sodium chloride, water
	Evicel (RxList, Bucharest, Romania)	Human fibrinogen, arginine hydrochloride, glycine, sodium chloride, sodium citrate, calcium chloride, human thrombin, human albumin, mannitol, sodium acetate, water
Cyanoacrylic Glue	Histoacryl (B. Braun, Bucharest, Romania)	Monomeric N-butyl-2-cyanoacrylate
	Glubran-2 (GEM Srl, Bucharest, Romania)	N-butyl-2-cyanoacrylate, Monomeric N-butyl-2-cyanoacrylate

**Table 10:** Tack fixation devices available on the market [115].

Product (Manufacturer)	Type of Tack	Material	Penetration Length	Resorption
ProTack (MedTronic, Bucharest, Romania)	Helical titanium tack	Titanium	3–4 mm	Non-absorbable
PermaFix (Bard, Bucharest, Romania)	Helical nontitanium	Polyacetal	6.7 mm	Non-absorbable
AbsorbaTack (Medtronic, Bucharest, Romania)	Absorbable	Poly(glycolide-co-L-lactide)	5.1 mm	3–5 months (complete 12 months)
PermaSorb (Bard, Bucharest, Romania)	Absorbable	Poly(D,L)-lactide	6.4 mm	16 months
SorbaFix (Bard, Bucharest, Romania)	Absorbable	Poly(D,L)-lactide	6.7 mm	12 months

**Table 11:** List of sutures available on the market (modified from reference [87]).

Product	Manufacturer	Type of Suture	Material
Nylon Ethilon	Dolphin Sutures Ethicon, Bucharest, Romania	Non-absorbable	Polyamide
Prolene Surgipro	J&J, Ethicon Covidien, Bucharest, Romania	Non-absorbable	Polypropylene
Ti-Cron	Covidien, Bucharest, Romania	Non-absorbable	Polyester
Monocryl	J&J Ethicon, Bucharest, Romania	Absorbable	Poliglecaprone
Polysorb	Covidien, Bucharest, Romania	Absorbable	Lactomer^TM^
Vicryl	J&J, Orion Sutures, Bucharest, Romania	Absorbable	Polyglactin
Dexon	N.A, Medtronic, Bucharest, Romania	Absorbable	Polyglycolic acid
Maxon	Medtronic, Bucharest, Romania	Absorbable	Polyglyconate
PDS	J&J, Ethicon, Bucharest, Romania	Absorbable	Polydioxanone

**Table 12:** Comparison of the most commonly used techniques [158,159,160,161].

Technique	Advantages	Disadvantages
Open (Lichtenstein)	- Low cost - Performed under local anesthesia - Short learning curve	- Higher postoperative pain - Longer recovery - Larger incision - Higher chronic pain risk
TAPP	- Excellent anatomical visualization - Suitable for bilateral/recurrent hernias - Easier for laparoscopic-trained surgeons	- Entry into peritoneal cavity (risk of visceral injury) - Longer operative time - Risk of postoperative ileus
TEP	- Avoids peritoneal entry - Lower risk of adhesions - Less postoperative pain in some studies	- Steeper learning curve - Limited working space - Technically demanding in obese or previously operated patients
SIL-TEP	- Better cosmetic outcome - Potentially less postoperative pain - Single incision	- Very steep learning curve - Limited triangulation - Not widely available
Robotic	- Enhanced dexterity and 3D vision - Ergonomic for surgeon - Useful in complex or recurrent cases	- High cost - Longer operative time - Limited availability - Not clearly superior in outcomes

**Table 13:** Long-term outcomes.

Technique/Mesh Type	Chronic Pain	Recurrence Rate	Foreign-Body Sensation	References
Open mesh (Lichtenstein)	Up to ~15%	~1.8–2.1% (5–10 y)	Moderate–high	[159]
Lightweight mesh (open/laparoscopic)	Reduced vs. HW mesh	Similar to HW	Low	[160]
TEP + partially absorbable mesh	0%	0.67% at 5 y	None reported	[161]
r-TAPP + mesh	Low	~0.5%	Low	[162]

## Data Availability

Data sharing is not applicable to this article.

## References

[1] Aydin M., Fikatas P., Denecke C., Pratschke J., Raakow J. (2021). Cost analysis of inguinal hernia repair: The influence of clinical and hernia-specific factors. Hernia.

[2] Zhang J., Wang J., Han X., Fan J., Huang C., Dong Y. (2025). Global burden and future trends of inguinal, femoral, and abdominal hernia in older adults: A systematic analysis from the Global Burden of Disease Study 2021. PLoS ONE.

[3] Maresova P., Peteja M., Lerch M., Zonca P., Kuca K. (2016). Costs of inguinal hernia repair associated with using different medical devices in the Czech Republic. Ther. Clin. Risk Manag..

[4] Garofil N.D., Bratucu M.N., Zurzu M., Paic V., Tigora A., Prunoiu V., Rogobete A., Balan A., Vladescu C., Strambu V.D.E. (2023). Groin Hernia Repair during the COVID-19 Pandemic-A Romanian Nationwide Analysis. Medicina.

[5] HerniaSurge Group (2018). International guidelines for groin hernia management. Hernia.

[6] Lockhart K., Dunn D., Teo S., Ng J.Y., Dhillon M., Teo E., van Driel M.L. (2018). Mesh versus non-mesh for inguinal and femoral hernia repair. Cochrane Database Syst. Rev..

[7] Fischer J.P., Basta M.N., Mirzabeigi M.N., Bauder A.R., Fox J.P., Drebin J.A., Serletti J.M., Kovach S.J. (2016). A Risk Model and Cost Analysis of Incisional Hernia After Elective, Abdominal Surgery Based Upon 12,373 Cases: The Case for Targeted Prophylactic Intervention. Ann. Surg..

[8] Flum D.R., Horvath K., Koepsell T. (2003). Have outcomes of incisional hernia repair improved with time?: A population-based analysis. Ann. Surg..

[9] Bassini E. (1887). Nuovo metodo per la cura radicale dell’ernia inguinale. Atti. Congr. Assoc. Med. Ital..

[10] Dulucq J.L., Wintringer P., Mahajna A. (2009). Laparoscopic totally extraperitoneal inguinal hernia repair: Lessons learned from 3100 hernia repairs over 15 years. Surg. Endosc..

[11] Huerta S., Timmerman C., Argo M., Favela J., Pham T., Kukreja S., Yan J., Zhu H. (2019). Open, Laparoscopic, and Robotic Inguinal Hernia Repair: Outcomes and Predictors of Complications. J. Surg. Res..

[12] Prabhu A.S., Carbonell A., Hope W., Warren J., Higgins R., Jacob B., Blatnik J., Haskins I., Alkhatib H., Tastaldi L. (2020). Robotic Inguinal vs Transabdominal Laparoscopic Inguinal Hernia Repair: The RIVAL Randomized Clinical Trial. JAMA Surg..

[13] de’Angelis N., Schena C.A., Moszkowicz D., Kuperas C., Fara R., Gaujoux S., Gillion J.F., Gronnier C., Loriau J., Mathonnet M. (2024). Robotic surgery for inguinal and ventral hernia repair: A systematic review and meta-analysis. Surg. Endosc..

[14] Anoldo P., Manigrasso M., D’Amore A., Musella M., De Palma G.D., Milone M. (2024). Abdominal Wall Hernias-State of the Art of Laparoscopic versus Robotic Surgery. J. Pers. Med..

[15] Kockerling F. (2017). Data and outcome of inguinal hernia repair in hernia registers—A review of the literature. Innov. Surg. Sci..

[16] Deveci C.D., Oberg S., Rosenberg J. (2023). Definition of Mesh Weight and Pore Size in Groin Hernia Repair: A Systematic Scoping Review of Randomised Controlled Trials. J. Abdom. Wall Surg. JAWS.

[17] Calomino N., Poto G.E., Carbone L., Micheletti G., Gjoka M., Giovine G., Sepe B., Bagnacci G., Piccioni S.A., Cuomo R. (2024). Weighing the benefits: Exploring the differential effects of light-weight and heavy-weight polypropylene meshes in inguinal hernia repair in a retrospective cohort study. Am. J. Surg..

[18] Najm A., Niculescu A.G., Gaspar B.S., Grumezescu A.M., Beuran M. (2023). A Review of Abdominal Meshes for Hernia Repair-Current Status and Emerging Solutions. Materials.

[19] Cobb W.S., Carbonell A.M., Kalbaugh C.L., Jones Y., Lokey J.S. (2009). Infection risk of open placement of intraperitoneal composite mesh. Am. Surg..

[20] Jeroukhimov I., Dykman D., Hershkovitz Y., Poluksht N., Nesterenko V., Yehuda A.B., Stephansky A., Zmora O. (2023). Chronic pain following totally extra-peritoneal inguinal hernia repair: A randomized clinical trial comparing glue and absorbable tackers. Langenbeck’s Arch. Surg..

[21] Trisca R., Oprea V., Toma M., Bucuri C.E., Stancu B., Grad O., Gherman C. (2024). The Effectiveness of Cyanoacrylates versus Sutures for Mesh Fixation after Lichtenstein Repair (SCyMeLi STUDY) A Systematic Review and Meta-Analyze of Randomized Controlled Trials. Chirurgia.

[22] Dams A., Vankeirsbilck J., Poelmans S., Kerschaever I., Borreman P., Berwouts L., De Mulder W., Colle J., Beunis A., Dhooghe V. (2023). Cyanoacrylate mesh fixation for laparoscopic inguinal hernia repair: A prospective, multicenter, single-arm study. Surg. Endosc..

[23] Vierstraete M., Chastan P., Meneghin A., Muysoms F. (2023). History of the Creation of Self-Gripping Mesh. J. Abdom. Wall Surg. JAWS.

[24] Lv Y., Yang B., Hao G., Wang Y. (2024). Mesh Fixation Versus Nonfixation in Laparoscopic Inguinal Hernia Repair: A Systematic Review and Meta-Analysis. Am. Surg..

[25] Zhang C., Li J., Suo H., Bai J. (2024). Non-fixation versus fixation of mesh in laparoscopic transabdominal preperitoneal repair of inguinal hernia: A systematic review and meta-analysis of randomized controlled trials. PLoS ONE.

[26] Stabilini C., van Veenendaal N., Aasvang E., Agresta F., Aufenacker T., Berrevoet F., Burgmans I., Chen D., de Beaux A., East B. (2023). Update of the international HerniaSurge guidelines for groin hernia management. BJS Open.

[27] Dönmez A.E., Goswami A.G., Raheja A., Bhadani A., El Kady A.E.S., Alniemi A., Awad A., Aladl A., Younis A., Alwali A. (2024). Access to and quality of elective care: A prospective cohort study using hernia surgery as a tracer condition in 83 countries. Lancet Glob. Health.

[28] Wang H., Wang X. (2024). Laparoscopic Versus Open Hernia Repair for Indirect Inguinal Hernia in Adolescents: A Retrospective Cohort Study. J. Investig. Surg..

[29] Picciochi M., Alexander P.V., Anyomih T., Boumas N., Crawford R., Enoch Gyamfi F., Hopane N., Isiagi M., Kamarajah S., Ledda V. (2025). Provision of inguinal hernia surgery in first-referral hospitals across low-and middle-income countries: Secondary analysis of an international cohort study. World J. Surg..

[30] Abedizadeh R., Majidi F., Khorasani H.R., Abedi H., Sabour D. (2024). Colorectal cancer: A comprehensive review of carcinogenesis, diagnosis, and novel strategies for classified treatments. Cancer Metastasis Rev..

[31] Lau W.Y. (2002). History of treatment of groin hernia. World J. Surg..

[32] Kingsorth A., Sanders D.L., LeBlanc K.A., Kingsnorth A., Sanders D.L. (2018). General Introduction and History of Hernia Surgery. Management of Abdominal Hernias.

[33] Patino J.F., Nyhus L.M., Condon R.E. (1995). A history of the treatment of hernia. Hernia.

[34] Furtado M., Claus C.M.P., Cavazzola L.T., Malcher F., Bakonyi-Neto A., Saad-Hossne R. (2019). Systemization of Laparoscopic Inguinal Hernia Repair (Tapp) Based on a New Anatomical Concept: Inverted Y and Five Triangles. Arq. Bras. Cir. Dig..

[35] Haeger K. (1989). The Illustrated History of Surgery.

[36] Paré A. (1953). The Apologie and Treatise of Ambroise Paré Containing the Voyages Made into Divers Places, with Many of His Writings upon Surgery. Yale J. Biol. Med..

[37] Nover A., Kümmel W.F. (1984). Surgeon and eye doctor Caspar Stromayr and the ophthalmological illustrations in his “Practica copiosa” (1559). Klin. Monatsblatter Augenheilkd..

[38] Cooper A.P. (1827). Lectures on the Principles and Practice of Surgery.

[39] Colles A. (1811). Treatise on Surgical Anatomy.

[40] Cloquet J., Wantz G.E. (1817). Recherches Anatomiques sur les Hernies de l’abdomen.

[41] Margotta R. (1968). An Illustrated History of Medicine.

[42] Ramjist J.K., Dossa F., Stukel T.A., Urbach D.R., Fu L., Baxter N.N. (2019). Reoperation for inguinal hernia recurrence in Ontario: A population-based study. Hernia.

[43] McArthur L.L. (1901). Autoplastic sutures in hernia and other diastases. JAMA.

[44] Devlin H.B. (1988). Management of Abdominal Hernias.

[45] Mair G.B. (1945). Preliminary report on the use of whole skin-grafts as a substitute for fascial sutures in the treatment of herniae. J. Br. Surg..

[46] Baylón K., Rodríguez-Camarillo P., Elías-Zúñiga A., Díaz-Elizondo J.A., Gilkerson R., Lozano K. (2017). Past, present and future of surgical meshes: A review. Membranes.

[47] Cumberland V. (1952). A preliminary report on the use of prefabricated nylon weave in the repair of ventral hernia. Med. J. Aust..

[48] Scales J. (1953). Tissue reactions to synthetic materials. Proc. R Soc. Med..

[49] Lichtenstein I.L., Shulman A.G., Amid P.K., Montllor M.M. (1989). The tension-free hernioplasty. Am. J. Surg..

[50] Ger R., Monroe K., Duvivier R., Mishrick A. (1990). Management of indirect inguinal hernias by laparoscopic closure of the neck of the sac. Am. J. Surg..

[51] Iossa A., Traumueller Tamagnini G., De Angelis F., Micalizzi A., Lelli G., Cavallaro G. (2024). TEP or TAPP: Who, when, and how?. Front. Surg..

[52] Srivastava N.K., Yadav A.S., Sinha R. (2023). A Comparative Evaluation of Extended Total Extraperitoneal Repair Versus Standard Total Extraperitoneal Repair and Transabdominal Preperitoneal Repair of Inguinal Hernias. J. Soc. Laparoendosc. Surg..

[53] Xie J., Koo D.C., Lee M.J., Sugiyama G. (2024). The evolution of minimally invasive inguinal hernia repairs. Ann. Laparosc. Endosc. Surg..

[54] Andresen K., Rosenberg J. (2024). Transabdominal pre-peritoneal (TAPP) versus totally extraperitoneal (TEP) laparoscopic techniques for inguinal hernia repair. Cochrane Database Syst. Rev..

[55] Mita K., Kobayashi N., Takahashi K., Sakai T., Shimaguchi M., Kouno M., Toyota N., Hatano M., Toyota T., Sasaki J. (2024). Anatomical recognition of dissection layers, nerves, vas deferens, and microvessels using artificial intelligence during transabdominal preperitoneal inguinal hernia repair. Hernia.

[56] Correia de Sa T., Jacome F., Basto T., Costa M., Goncalves A., Teixeira N., Castro Neves L., Barros da Silva J. (2024). Transabdominal preperitoneal (TAPP) repair for emergency groin hernia: A systematic review. Hernia.

[57] Sivakumar J., Chen Q., Hii M.W., Cullinan M., Choi J., Steven M., Crosthwaite G. (2023). Learning curve of laparoscopic inguinal hernia repair: Systematic review, meta-analysis, and meta-regression. Surg. Endosc..

[58] Peethambaran M.S., Rajendran R.R., Murthy N.G. (2024). Totally Extraperitoneal Repair of Grynfeltt’s Hernia: Easy Solution for a Rare Problem. Cureus.

[59] Baginski B., Tran D., Ogola G., Arnold D. (2024). A single-center retrospective review of laparoscopic totally extraperitoneal versus robotic transabdominal preperitoneal inguinal hernia repair. Proceedings.

[60] Basukala S., Shrestha O., Chhetri S.T., Thapa N., Oli S., Mehta B.K., Pokhrel N., Tiwari B. (2024). Transabdominal Preperitoneal (TAPP) Compared to Totally Extraperitoneal (TEP) for Primary Inguinal Hernia Repair in a Military Hospital: A Retrospective Cohort Study. Health Sci. Rep..

[61] Ozel Y., Kara Y.B. (2024). Comparison of Clinical Outcomes of Laparoscopic Totally Extraperitoneal (TEP) and Transabdominal Preperitoneal (TAPP) Techniques in Bilateral Inguinal Hernia Repair: A Retrospective Study. Cureus.

[62] Dokania M.K., Ankur A., Agarwal N., Jain A., Anshu A., Singh R.A.K. (2024). Comparison of Perioperative Complication Rates of Total Extraperitoneal and Transabdominal Preperitoneal Repairs in Primary Inguinal Hernia. J. West Afr. Coll. Surg..

[63] Ielpo B., Nunez-Alfonsel J., Duran H., Diaz E., Fabra I., Caruso R., Malave L., Ferri V., Barzola E., Quijano Y. (2018). Cost-effectiveness of Randomized Study of Laparoscopic Versus Open Bilateral Inguinal Hernia Repair. Ann. Surg..

[64] Senol Z. (2025). Evaluation of Surgical Results and Effectiveness of Laparoscopic Transabdominal Preperitoneal and Laparoscopic Totally Extraperitoneal Approaches in Bilateral Inguinal Hernia Repair: A Randomized Analysis. J. Laparoendosc. Adv. Surg. Tech. Part A.

[65] Almutairi H., Alshammari R.S., Alharbi M.J., Althobaiti D.M., Alghamdi R.S., Alsamiri S., Mawash S.W., Ahmed D.A., Alamoudi A.A., Arif F.Y. (2024). Laparoscopic Management of Inguinal Hernia: A Systematic Review and Updated Network Meta-Analysis of Randomized Controlled Trials. Cureus.

[66] Rivas J.F., Molina A.P.R.F., Carmona J.M. (2021). Transabdominal preperitoneal (TAPP) inguinal hernia repair: How we do it. Ann. Laparosc. Endosc. Surg..

[67] Carter J., Duh Q.Y. (2011). Laparoscopic repair of inguinal hernias. World J. Surg..

[68] Mubark M., Mohammed H.A., Mohamed M.A. (2023). Transabdominal pre-peritoneal (TAPP) versus totally extraperitoneal (TEP) laparoscopic techniques for inguinal hernia repair. SVU-Int. J. Med. Sci..

[69] Daes J., Felix E. (2017). Critical View of the Myopectineal Orifice. Ann. Surg..

[70] Sözen S., Erdem H. (2022). Hernia Surgery.

[71] Delibegović S. (2023). TEP, STEP BY STEP. South-East Eur. Endo-Surg. J..

[72] Ferzli G., Iskandar M. (2019). Laparoscopic totally extra-peritoneal (TEP) inguinal hernia repair. Ann. Laparosc. Endosc. Surg..

[73] Lomanto D., Goel R. (2012). Intraoperative Complications During Laparoscopic Hernia Repair. The SAGES Manual of Hernia Repai.

[74] Davis C.J., Arregui M.E. (2003). Laparoscopic repair for groin hernias. Surg. Clin..

[75] Felix E.L., Harbertson N., Vartanian S. (1999). Laparoscopic hernioplasty: Significant complications. Surg. Endosc..

[76] Sartori A., De Luca M., Noaro G., Piatto G., Pignata G., Di Leo A., Lauro E., Andreuccetti J. (2021). Rare intraoperative and postoperative complications after transabdominal laparoscopic hernia repair: Results from the multicenter wall hernia group registry. J. Laparoendosc. Adv. Surg. Tech..

[77] Frydenlund P., Ramaswamy A. (2018). Intraoperative and postoperative complications of MIS inguinal hernia repair. The SAGES Manual of Hernia Surgery.

[78] Poffenberger R.J. (1996). Laparoscopic repair of intraperitoneal bladder injury: A simple new technique. Urology.

[79] Tamme C., Scheidbach H., Hampe C., Schneider C., Köckerling F. (2003). Totally extraperitoneal endoscopic inguinal hernia repair (TEP). Surg. Endosc. Other Interv. Tech..

[80] Moreno-Egea A., Paredes P.G., Perello J.M., Campillo-Soto A., Baena E.G., Muñoz J.R.O., Aguayo-Albasini J.L. (2010). Vascular injury by tacks during totally extraperitoneal endoscopic inguinal hernioplasty. Surg. Laparosc. Endosc. Percutaneous Tech..

[81] Fitzgibbons R.J., Camps J., Cornet D.A., Nguyen N.X., Litke B.S., Annibali R., Salerno G.M. (1995). Laparoscopic inguinal herniorrhaphy results of a multicenter trial. Ann. Surg..

[82] Lipskar A.M., Reiner M.A. (2013). Cord Structure Complications in Inguinal Hernia Surgery.

[83] Ghariani W., Dougaz M.W., Jerraya H., Khalfallah M., Bouasker I., Dziri C. (2019). Recurrence Factors of Groin Hernia: A systematic Review. La Tunis. Medicale.

[84] Stan A.-M., Preda S.-D., Râmboiu S., Câråu D., Pãtraæcu Æ., Cazacu S., Biciuæcã V., Ætiolicã A.T., Andronic O., Pãduraru D.N. (2023). Ventral Hernia Repair and Drainage–A Prospective, Observational, and Comparative Study of Outcomes. Chirurgia.

[85] Van Veen R., Wijsmuller A., Vrijland W., Hop W., Lange J., Jeekel J. (2007). Long-term follow-up of a randomized clinical trial of non-mesh versus mesh repair of primary inguinal hernia. J. Br. Surg..

[86] Bittner R., Leibl B., Kraft B., Schwarz J. (2011). One-year results of a prospective, randomised clinical trial comparing four meshes in laparoscopic inguinal hernia repair (TAPP). Hernia.

[87] See C.W., Kim T., Zhu D. (2020). Hernia mesh and hernia repair: A review. Eng. Regen..

[88] Hollinsky C., Sandberg S., Koch T., Seidler S. (2008). Biomechanical properties of lightweight versus heavyweight meshes for laparoscopic inguinal hernia repair and their impact on recurrence rates. Surg. Endosc..

[89] Idrees S., Jindal S., Gupta M., Sarangi R. (2018). Surgical meshes–The search continues. Curr. Med. Res. Pract..

[90] Cobb W.S., Burns J.M., Kercher K.W., Matthews B.D., Norton H.J., Heniford B.T. (2005). Normal intraabdominal pressure in healthy adults. J. Surg. Res..

[91] Kalaba S., Gerhard E., Winder J.S., Pauli E.M., Haluck R.S., Yang J. (2016). Design strategies and applications of biomaterials and devices for hernia repair. Bioact. Mater..

[92] Brown C., Finch J. (2010). Which mesh for hernia repair?. Ann. R. Coll. Surg. Engl..

[93] Earle D.B., Mark L.A. (2008). Prosthetic material in inguinal hernia repair: How do I choose?. Surg. Clin. N. Am..

[94] Li J., Ji Z., Cheng T. (2012). Lightweight versus heavyweight in inguinal hernia repair: A meta-analysis. Hernia.

[95] O’dwyer P., Kingsnorth A., Molloy R., Small P., Lammers B., Horeyseck G. (2005). Randomized clinical trial assessing impact of a lightweight or heavyweight mesh on chronic pain after inguinal hernia repair. J. Br. Surg..

[96] Melkemichel M., Bringman S., Nilsson H., Widhe B. (2020). Patient-reported chronic pain after open inguinal hernia repair with lightweight or heavyweight mesh: A prospective, patient-reported outcomes study. J. Br. Surg..

[97] Bona S., Rosati R., Opocher E., Fiore B., Montorsi M. (2018). Pain and quality of life after inguinal hernia surgery: A multicenter randomized controlled trial comparing lightweight vs heavyweight mesh (Supermesh Study). Updates Surg..

[98] Bakker W.J., Aufenacker T.J., Boschman J.S., Burgmans J.P. (2021). Heavyweight mesh is superior to lightweight mesh in laparo-endoscopic inguinal hernia repair: A meta-analysis and trial sequential analysis of randomized controlled trials. Ann. Surg..

[99] DeBord J.R. (1998). The historical development of prosthetics in hernia surgery. Surg. Clin. N. Am..

[100] Amid P. (1997). Classification of biomaterials and their related complications in abdominal wall hernia surgery. Hernia.

[101] Trandafir A.F., Popa D.E., Vasile D. (2017). Prostheses Used in Laparoscopic Inguinal Hernia Repair: Biocompatibility, Postoperative Complications and Quality of Life–Review of the Literature. Maedica.

[102] Gavlin A., Kierans A.S., Chen J., Song C., Guniganti P., Mazzariol F.S. (2020). Imaging and treatment of complications of abdominal and pelvic mesh repair. Radiographics.

[103] Spotnitz W.D., Falstrom J.K., Rodeheaver G.T. (1997). The role of sutures and fibrin sealant in wound healing. Surg. Clin. N. Am..

[104] Matthew T.L., Spotnitz W.D., Kron I.L., Daniel T.M., Tribble C.G., Nolan S.P. (1990). Four years’ experience with fibrin sealant in thoracic and cardiovascular surgery. Ann. Thorac. Surg..

[105] Byrne D.J., Hardy J., Wood R.A., McIntosh R., Cuschieri A. (1991). Effect of fibrin glues on the mechanical properties of healing wounds. Br. J. Surg..

[106] Katkhouda N., Mavor E., Friedlander M.H., Mason R.J., Kiyabu M., Grant S.W., Achanta K., Kirkman E.L., Narayanan K., Essani R. (2001). Use of fibrin sealant for prosthetic mesh fixation in laparoscopic extraperitoneal inguinal hernia repair. Ann. Surg..

[107] Mohammadi Tofigh A., Karimian Ghadim M., Bohlooli M. (2021). Comparing suture with N-Hexyl Cyanoacrylate glue for mesh fixation in inguinal hernia repair, a randomised clinical trial. Am. J. Surg..

[108] Mahardawi B., Jiaranuchart S., Rochanavibhata S., Siriwat K., Mattheos N., Pimkhaokham A. (2024). Cyanoacrylate tissue adhesive versus silk sutures for mandibular third molar surgery: A systematic review and meta-analysis. Clin. Oral Investig..

[109] Giray C.B., Atasever A., Durgun B., Araz K. (1997). Clinical and electron microscope comparison of silk sutures and n-butyl-2-cyanoacrylate in human musosa. Aust. Dent. J..

[110] Ayyıldız S.N., Ayyıldız A. (2017). Cyanoacrylic tissue glues: Biochemical properties and their usage in urology. Urkish J. Urol..

[111] Burati M., Scaini A., Fumagalli L.A., Gabrielli F., Chiarelli M. (2019). Mesh fixation methods in groin hernia surgery. Techniques and Innovation in Hernia Surgery.

[112] Rancke-Madsen P., Oberg S., Rosenberg J. (2025). Mesh fixation in laparoscopic groin hernia repair: A comprehensive review of techniques and devices. Hernia.

[113] Wirth U., Saller M.L., von Ahnen T., Kockerling F., Schardey H.M., Schopf S. (2020). Long-term outcome and chronic pain in atraumatic fibrin glue versus staple fixation of extra light titanized meshes in laparoscopic inguinal hernia repair (TAPP): A single-center experience. Surg. Endosc..

[114] Smith A.M., Faulkner J.D., Chase N., Eckhauser F.E., Hope W.W. (2021). The Effect of Tack Fixation Methods on Outcomes in Laparoscopic Ventral Hernia Repair. J. Laparoendosc. Adv. Surg. Tech. Part A.

[115] Reynvoet E., Berrevoet F. (2014). Pros and cons of tacking in laparoscopic hernia repair. Surg. Technol. Int..

[116] LeBlanc K.A. (2003). Tack hernia: A new entity. J. Soc. Laparoendosc. Surg..

[117] Firestone D.E., Lauder A.J. (2010). Chemistry and mechanics of commonly used sutures and needles. J. Hand Surg..

[118] Yag-Howard C. (2014). Sutures, needles, and tissue adhesives: A review for dermatologic surgery. Dermatol. Surg..

[119] Moy R.L., Waldman B., Hein D.W. (1992). A review of sutures and suturing techniques. J. Dermatol. Surg. Oncol..

[120] Lee Y.J., Kim J.H., Kim C.H., Lee G.R., Lee Y.S., Kim H.J. (2021). Single incision laparoscopic totally extraperitoneal hernioplasty: Lessons learned from 1,231 procedures. Ann. Surg. Treat. Res..

[121] Cugura J.F., Kirac I., Kulis T., Jankovic J., Beslin M.B. (2008). First case of single incision laparoscopic surgery for totally extraperitoneal inguinal hernia repair. Acta Clin. Croat..

[122] Kim J.H., An C.H., Lee Y.S., Kim H.Y., Lee J.I. (2015). Single incision laparoscopic totally extraperitoneal hernioplasty (SIL-TEP): Experience of 512 procedures. Hernia.

[123] Escobar Dominguez J.E., Gonzalez A., Donkor C. (2015). Robotic inguinal hernia repair. J. Surg. Oncol..

[124] Chen Y.J., Huynh D., Nguyen S., Chin E., Divino C., Zhang L. (2017). Outcomes of robot-assisted versus laparoscopic repair of small-sized ventral hernias. Surg. Endosc..

[125] Waite K.E., Herman M.A., Doyle P.J. (2016). Comparison of robotic versus laparoscopic transabdominal preperitoneal (TAPP) inguinal hernia repair. J. Robot. Surg..

[126] Kolachalam R., Dickens E., D’Amico L., Richardson C., Rabaza J., Gamagami R., Gonzalez A. (2018). Early outcomes of robotic-assisted inguinal hernia repair in obese patients: A multi-institutional, retrospective study. Surg. Endosc..

[127] Charles E.J., Mehaffey J.H., Tache-Leon C.A., Hallowell P.T., Sawyer R.G., Yang Z. (2018). Inguinal hernia repair: Is there a benefit to using the robot?. Surg. Endosc..

[128] Xie H., Jia Y., Liu S. (2024). Integration of artificial intelligence in clinical laboratory medicine: Advancements and challenges. Interdiscip. Med..

[129] Abdelmoaty W.F., Dunst C.M., Neighorn C., Swanstrom L.L., Hammill C.W. (2019). Robotic-assisted versus laparoscopic unilateral inguinal hernia repair: A comprehensive cost analysis. Surg. Endosc..

[130] Huerta S., Garza A.M. (2025). A Systematic Review of Open, Laparoscopic, and Robotic Inguinal Hernia Repair: Management of Inguinal Hernias in the 21st Century. J. Clin. Med..

[131] Sabbatini F., La Regina D., Murgante Testa N., Senatore A.M., Saporito A., Pini R., Mongelli F. (2024). Hospital costs of robotic-assisted and open treatment of large ventral hernias. Sci. Rep..

[132] Hung T.Y., Wu C.C., Chen L.S., Kang Y.N. (2020). Safety of two common laparoscopic inguinal herniorrhaphy approaches: An updated systematic review with meta-analysis of randomized clinical trials. Transl. Androl. Urol..

[133] Aiolfi A., Cavalli M., Del Ferraro S., Manfredini L., Lombardo F., Bonitta G., Bruni P.G., Panizzo V., Campanelli G., Bona D. (2021). Total extraperitoneal (TEP) versus laparoscopic transabdominal preperitoneal (TAPP) hernioplasty: Systematic review and trial sequential analysis of randomized controlled trials. Hernia.

[134] Bracale U., Melillo P., Pignata G., Di Salvo E., Rovani M., Merola G., Pecchia L. (2012). Which is the best laparoscopic approach for inguinal hernia repair: TEP or TAPP? A systematic review of the literature with a network meta-analysis. Surg. Endosc..

[135] Löfgren J., Nordin P., Ibingira C., Matovu A., Galiwango E., Wladis A. (2016). A Randomized Trial of Low-Cost Mesh in Groin Hernia Repair. N. Engl. J. Med..

[136] Popa F., Rosca O., Georgescu A., Cannistra C. (2016). Reconstruction of the Abdominal Wall in Anatomical Plans. Pre- and Postoperative Keys in Repairing “Cold” Incisional Hernias. Clujul Med..

[137] Ünek T., Sökmen S., Egeli T., Avkan Oğuz V., Ellidokuz H., Obuz F. (2019). The results of expanded-polytetrafluoroethylene mesh repair in difficult abdominal wall defects. Asian J. Surg..

[138] Koehler R.H., Begos D., Berger D., Carey S., LeBlanc K., Park A., Ramshaw B., Smoot R., Voeller G. (2003). Minimal adhesions to ePTFE mesh after laparoscopic ventral incisional hernia repair: Reoperative findings in 65 cases. J. Soc. Laparoendosc. Surg..

[139] Aydemir Sezer U., Sanko V., Gulmez M., Aru B., Sayman E., Aktekin A., Vardar Aker F., Yanıkkaya Demirel G., Sezer S. (2019). Polypropylene composite hernia mesh with anti-adhesion layer composed of polycaprolactone and oxidized regenerated cellulose. Mater. Sci. Eng. C Mater. Biol. Appl..

[140] Sanbhal N., Saitaer X., Li Y., Mao Y., Zou T., Sun G., Wang L. (2018). Controlled Levofloxacin Release and Antibacterial Properties of β-Cyclodextrins-Grafted Polypropylene Mesh Devices for Hernia Repair. Polymers.

[141] Pérez-Köhler B., Benito-Martínez S., Rodríguez M., García-Moreno F., Pascual G., Bellón J.M. (2019). Experimental study on the use of a chlorhexidine-loaded carboxymethylcellulose gel as antibacterial coating for hernia repair meshes. Hernia.

[142] Wang Y., Cao Z., Cheng R., Qin M., Zhang D., Deng L., Chen X., Cui W. (2019). Immunomodulated electrospun fibrous scaffolds via bFGF camouflage for pelvic regeneration. Appl. Mater. Today.

[143] Miao Y.-H., Wang X., Zhao X.-M., Hu Y.-W., Liu X., Deng D.-W. (2025). Co-assembly strategies of natural plant compounds for improving their bioavailability. Food Med. Homol..

[144] Zhu Y.X., Jia H.R., Jiang Y.W., Guo Y., Duan Q.Y., Xu K.F., Shan B.H., Liu X., Chen X., Wu F.G. (2024). A red blood cell-derived bionic microrobot capable of hierarchically adapting to five critical stages in systemic drug delivery. Exploration.

[145] Sanders D.L., Waydia S. (2014). A systematic review of randomised control trials assessing mesh fixation in open inguinal hernia repair. Hernia.

[146] Sajid M.S., Ladwa N., Kalra L., McFall M., Baig M.K., Sains P. (2013). A meta-analysis examining the use of tacker mesh fixation versus glue mesh fixation in laparoscopic inguinal hernia repair. Am. J. Surg..

[147] Fenger A.Q., Helvind N.M., Pommergaard H.C., Burcharth J., Rosenberg J. (2016). Fibrin sealant for mesh fixation in laparoscopic groin hernia repair does not increase long-term recurrence. Surg. Endosc..

[148] Helvind N.M., Andresen K., Rosenberg J. (2013). Lower reoperation rates with the use of fibrin sealant versus tacks for mesh fixation. Surg. Endosc..

[149] Pascual G., Mesa-Ciller C., Rodríguez M., Pérez-Köhler B., Gómez-Gil V. (2018). Pre-clinical assay of the tissue integration and mechanical adhesion of several types of cyanoacrylate adhesives in the fixation of lightweight polypropylene meshes for abdominal hernia repair. PLoS ONE.

[150] Agarwal P.K., Sutrave T., Kaushal D., Vidua R., Malik R., Maurya A.P. (2023). Comparison of Postoperative Chronic Groin Pain After Repair of Inguinal Hernia Using Nonabsorbable Versus Absorbable Sutures for Mesh Fixation. Cureus.

[151] Bilaloglu M.H. (2024). The effect of absorbable vs. non-absorbable fixation on early complications in laparoscopic transabdominal preperitoneal (TAPP) inguinal herniorrhaphy. Ann. Med. Res..

[152] Khan R.M.A., Bughio M., Ali B., Hajibandeh S., Hajibandeh S. (2018). Absorbable versus non-absorbable tacks for mesh fixation in laparoscopic ventral hernia repair: A systematic review and meta-analysis. Int. J. Surg..

[153] Sajid M.S., McFall M.R., Whitehouse P.A., Sains P.S. (2014). Systematic review of absorbable vs non-absorbable sutures used for the closure of surgical incisions. World J. Gastrointest. Surg..

[154] Radu P., Brătucu M., Garofil D., Pasnicu C., Iorga C., Popa F., Strâmbu V. (2013). Molecular factors of failure in incisional hernia surgery. Chirurgia.

[155] Klosterhalfen B., Junge K., Klinge U. (2005). The lightweight and large porous mesh concept for hernia repair. Expert Rev. Med. Devices.

[156] Radu P., Brătucu M., Garofil D., Goleanu V., Popa F., Strâmbu V. (2015). The Role of Collagen Metabolism in the Formation and Relapse of Incisional Hernia. Chirurgia.

[157] Parker S.G., Mallett S., Quinn L., Wood C.P.J., Boulton R.W., Jamshaid S., Erotocritou M., Gowda S., Collier W., Plumb A.A.O. (2021). Identifying predictors of ventral hernia recurrence: Systematic review and meta-analysis. BJS Open.

[158] Arrey E., Young T., Alford A. (2024). A Comprehensive Review of the Evolution of Minimally Invasive Hernia Repair: Historical Milestones to Modern Clinical Practice. Curr. Surg. Rep..

[159] McCormack K., Wake B., Perez J., Fraser C., Cook J., McIntosh E., Vale L., Grant A. (2005). Laparoscopic surgery for inguinal hernia repair: Systematic review of effectiveness and economic evaluation. Health Technol. Assess..

[160] Li X., Li Y.-J., Dong H., Wang D.-C., Wei J. (2024). Meta-analysis of the effectiveness and safety of robotic-assisted versus laparoscopic transabdominal preperitoneal repair for inguinal hernia. PLoS ONE.

[161] Simons M.P., Aufenacker T., Bay-Nielsen M., Bouillot J.L., Campanelli G., Conze J., de Lange D., Fortelny R., Heikkinen T., Kingsnorth A. (2009). European Hernia Society guidelines on the treatment of inguinal hernia in adult patients. Hernia.

[162] Lund H., Spanager L., Winther A.C.R., Gierløff M., Sunekær K., Kleif J., Bertelsen C.A. (2025). Recurrence and complications after laparoscopic inguinal hernia repair using a self-adherent mesh: A patient-reported follow-up study. Surg. Endosc..

[163] Nikkolo C., Lillsaar T., Vaasna T., Kirsimägi Ü., Lepner U. (2025). Ten-Year Results of Inguinal Hernia Open Mesh Repair. J. Abdom. Wall Surg..

[164] Kabaoglu B., Sobutay E., Bilgic C. (2024). Postoperative Outcomes and Recurrence Rate in Laparoscopic Tep Inguinal Hernia Repairs Using Partially Absorbable Meshes: A Retrospective Single-Surgeon Study Over a 5-Year Period. Med. Bull. Sisli Etfal Hosp..

[165] Viera O.J., Florin J.L., Morales K.E. (2025). Long-term outcomes of robotic inguinal hernia repair (r-TAPP): A retrospective review of 434 consecutive cases by a single surgeon with 3–8 years of follow-up. J. Robot. Surg..

[166] Mulita F., Parchas N., Solou K., Tchabashvili L., Gatomati F., Iliopoulos F., Maroulis I. (2020). Postoperative pain scores after open inguinal hernia repair: Comparison of three postoperative analgesic regimens. Med. Arch..

[167] Coppens S., Gidts J., Huynen P., Van De Velde M., Joshi G. (2020). Pain management after open inguinal hernia repair: An updated systematic review and procedure-specific postoperative pain management (PROSPECT/ESRA) recommendations. Acta Anaesthesiol. Belg..

[168] Sahoo R.K., Pradhan A., Samanta P., Senapati L.K., Satapathy G.C. (2024). Effect of ultrasound-guided ilioinguinal-iliohypogastric nerve block on chronic pain in patients undergoing open inguinal hernia surgery under spinal anesthesia: A randomized double-blind study. Korean J. Pain.

